# When Does Model-Based Control Pay Off?

**DOI:** 10.1371/journal.pcbi.1005090

**Published:** 2016-08-26

**Authors:** Wouter Kool, Fiery A. Cushman, Samuel J. Gershman

**Affiliations:** 1 Department of Psychology, Harvard University, Cambridge, Massachusetts, United States of America; 2 Center for Brain Science, Harvard University, Cambridge, Massachusetts, United States of America; Oxford University, UNITED KINGDOM

## Abstract

Many accounts of decision making and reinforcement learning posit the existence of two distinct systems that control choice: a fast, automatic system and a slow, deliberative system. Recent research formalizes this distinction by mapping these systems to “model-free” and “model-based” strategies in reinforcement learning. Model-free strategies are computationally cheap, but sometimes inaccurate, because action values can be accessed by inspecting a look-up table constructed through trial-and-error. In contrast, model-based strategies compute action values through planning in a causal model of the environment, which is more accurate but also more cognitively demanding. It is assumed that this trade-off between accuracy and computational demand plays an important role in the arbitration between the two strategies, but we show that the hallmark task for dissociating model-free and model-based strategies, as well as several related variants, do not embody such a trade-off. We describe five factors that reduce the effectiveness of the model-based strategy on these tasks by reducing its accuracy in estimating reward outcomes and decreasing the importance of its choices. Based on these observations, we describe a version of the task that formally and empirically obtains an accuracy-demand trade-off between model-free and model-based strategies. Moreover, we show that human participants spontaneously increase their reliance on model-based control on this task, compared to the original paradigm. Our novel task and our computational analyses may prove important in subsequent empirical investigations of how humans balance accuracy and demand.

## Author Summary

When you make a choice about what groceries to get for dinner, you can rely on two different strategies. You can make your choice by relying on habit, simply buying the items you need to make a meal that is second nature to you. However, you can also plan your actions in a more deliberative way, realizing that the friend who will join you is a vegetarian, and therefore you should not make the burgers that have become a staple in your cooking. These two strategies differ in how *computationally demanding* and *accurate* they are. While the habitual strategy is less computationally demanding (costs less effort and time), the deliberative strategy is more accurate. Scientists have been able to study the distinction between these strategies using a task that allows them to measure how much people rely on habit and planning strategies. Interestingly, we have discovered that in this task, the deliberative strategy does not increase performance accuracy, and hence does not induce a trade-off between accuracy and demand. We describe why this happens, and improve the task so that it embodies an accuracy-demand trade-off, providing evidence for theories of cost-based arbitration between cognitive strategies.

## Introduction

Theoretical accounts of decision making emphasize a distinction between two systems competing for control of behavior [1–6]: one that is fast and automatic, and one that is slow and deliberative. These systems occupy different points along a trade-off between accuracy and computational demand (henceforth demand), making each one suitable for particular task demands. This raises the problem of arbitration: how does the brain adaptively determine which system to use at any given time? Answering this question depends on models and experimental tasks that embody the accuracy-demand trade-off at the heart of dual-system models.

Recent research formalizes the dual-system architecture in the framework of reinforcement learning [7, 8], a computational approach to value-guided decision-making that we describe in further detail below. The application of reinforcement learning methods to dual-process models of decision-making sparked an explosion of empirical and theoretical developments over the past decade because it offers a computationally precise characterization of the distinction between “automatic” and “controlled” processes for the task of value guided decision-making. Current research assumes that experimental methods grounded in reinforcement learning also capture a trade-off between accuracy (the proportion of value-maximizing actions) and computational demand (the minimization of computational effort and related costs), but this assumption remains largely untested (cf. [9]).

**Fig 1:**
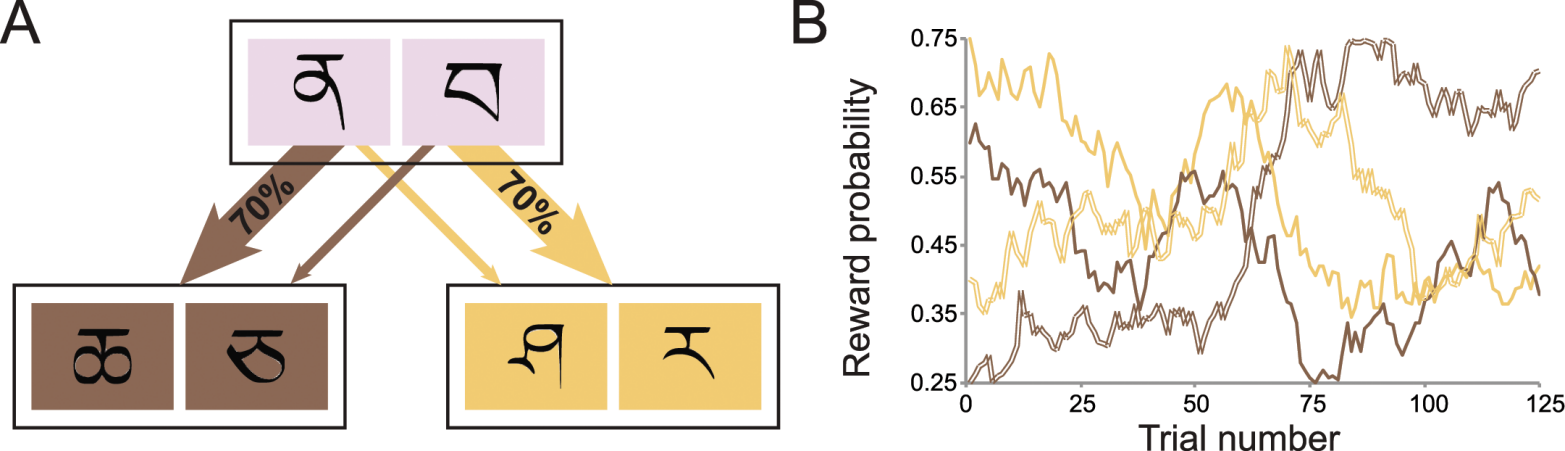
Design of the Daw two-step task. (A) State transition structure of the original two-step paradigm. Each first-stage choice has a high probability of transitioning to one of two states and a low probability of transitioning to the other. Each second-stage choice is associated with a probability of obtaining a binary reward. (B) To encourage learning, the second-stage reward probabilities change slowly over the course of the experiment.

Currently, the dominant method that aims to dissociate mechanisms of behavioral control within the reinforcement learning framework is the “two-step task” introduced by Daw, Gershman, Seymour, Dayan, and Dolan [8] (Fig 1A), which we describe in detail in the next section. This task has proven to be a useful and popular tool to characterize the neural [8, 10–18], behavioral [19–31] and clinical [32–35] implications of dual-process models within the reinforcement learning framework. However, in this paper we argue that the two-step task does not induce a trade-off between accuracy and demand: Our simulations show that the “deliberative” strategy does not increase performance accuracy on the task. These simulations mirror a recent report by Akam, Costa, and Dayan [9], who also show that the two-step task does not embody a trade-off between model-based control and reward. Here, we expand on that result by showing that it holds across an exhaustive range of reinforcement learning parameters. Furthermore, we show that the same shortcoming is present in other, more recent variants of the task that have been reported. We then identify five factors that collectively restrict the accuracy benefits posited to arise from model-based control. Finally, we describe a novel task that induces the accuracy-demand trade-off, while retaining the ability to dissociate formally between distinct processes of behavioral control.

### Dual-process models in the reinforcement learning setting

The fundamental problem in reinforcement learning is estimation of state-action values (cumulative future reward), which an agent then uses to choose actions. In the dual-system theory, the fast and automatic system corresponds to a “model-free” reinforcement learning strategy, which estimates state-action values from trial-and-error learning [36, 37]. In essence, this strategy is an elaborated version of Thorndike’s Law of Effect: actions that previously led to reward are more likely to be taken in the future. The strategy is “model-free” because it has no representation of the environment’s causal structure (i.e., the transition function between states and the reward function in each state). Instead, it incrementally constructs a look-up table or function approximation from which values can be quickly computed. However, this strategy can lead to errors if the environment changes, because the entire value function must be incrementally updated to accommodate changes. In addition, the strategy can produce sub-optimal credit assignment [8], a property we explore below. These forms of brittleness illustrate how model-free learning gives rise to “habits”—fast but inflexible response tendencies stamped in by repetition.

The slow and deliberative system corresponds to a “model-based” learning strategy that possesses operating characteristics complementary to the model-free strategy. This strategy learns an explicit causal model of the environment, which it uses to construct plans (e.g., by dynamic programming or tree search). In contrast to the habitual nature of the model-free strategy, the capacity to plan enables the model-based strategy to flexibly pursue goals. While more computationally expensive (hence slower and more effortful) than the model-free approach, it has the potential to be more accurate, because changes in the environment can be immediately incorporated into the model. The availability of a causal model also allows the model-based strategy to solve the credit-assignment problem optimally.

This dual-system framework sketched above can account for important findings in the reinforcement learning literature, such as insensitivity to outcome devaluation following overtraining of an action-reward contingency [7, 38]. Furthermore, the framework has spurred a wealth of new research on the neural [8, 10–13, 39, 40] and behavioral implications of competition and cooperation between reinforcement learning strategies [19–25, 29, 41, 42].

How might the brain arbitrate between model-free and model-based strategies? Since the model-based strategy attains more accurate performance through effortful computation, people can (up to a point) increase reward by engaging this system. However, in time-critical decision making settings, the model-based strategy may be too slow to be useful. Furthermore, if cognitive effort enters into the reward function [43–45], then it may be rational to prefer the model-free strategy in situations where the additional cognitive effort of model-based planning does not appreciably increase reward. It has been hypothesized that this trade-off between accuracy and demand plays a pivotal role in the arbitration between the two strategies [38, 46–50], but so far direct evidence for arbitration has been sparse [51].

## Methods

### The Daw two-step task

Here, we will first describe in detail the design of the Daw two-step task, and the reinforcement-learning model of this task [8]. Next, we will show through computational simulations that model-based planning on this task does not yield increased performance accuracy. Finally, we will discuss several factors that contribute to this shortcoming in the current approach in this two-step task, and related paradigms.

#### Experimental design

In the two-step task, participants make a series of choices between two stimuli, which lead probabilistically to one of two second-stage states (Fig 1A). These second-stage states require a choice between stimuli that offer different probabilities of obtaining monetary reward. To encourage learning, the reward probabilities of these second-stage choices change slowly and independently throughout the task (Fig 1B), according to a Gaussian random walk (mean = 0, *σ* = 0.025) with reflecting boundaries at 0.25 and 0.75. Crucially, each first-stage option leads more frequently (70%) to one of the second-stage states (a “common” transition), whereas it leads to the other state in a minority of the choices (a “rare” transition).

**Fig 2:**
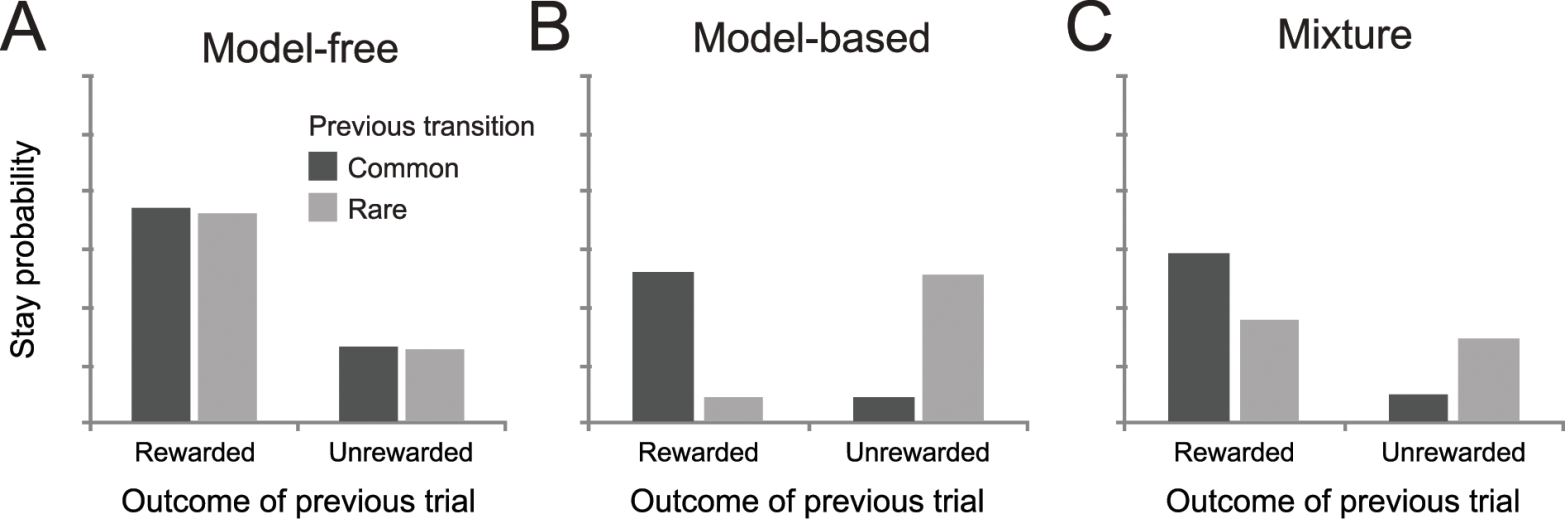
Probability of repeating the first-stage choice for three agents. (A) For model-free agents, the probability of repeating the previous choice is dependent only on whether a reward was obtained, and not on transition structure. (B) Model-based behavior is reflected in an interaction between previous transition and outcome, increasing the probability of transitioning to the state where reward was obtained. (C) Behavioral performance on this task reflects features of both model-based and model-free decision making, the main effect of previous reward and its interaction with the previous transition.

These low-probability transitions allow for a behavioral dissociation between habitual and goal-directed choice. Since the model-free strategy is insensitive to the structure the task, it will simply increase the likelihood of performing an action if it previously led to reward, regardless whether this reward was obtained after a common or rare transition. Choice dictated by the model-based strategy, on the other hand, reflects an interaction between the transition type and reward on the previous trial (Fig 2A). This strategy will decrease the tendency of repeating a first-stage action after a reward and a rare transition, since the alternative first-stage action is more likely to lead to the previously rewarded second-stage state (Fig 2B). Empirically, behavioral performance on this task reflects a mixture of these two strategies (Fig 2C). That is, the stay probability shows a main effect of reward, increasing when the previous trial was rewarded, but also shows the model-based crossover interaction between the previous transition and reward.

#### Computational model

Behavior on the Daw two-step task can be modeled using an established dual-system reinforcement-learning model [7, 8, 40]. The task consists of three states across two stages (first stage: *s*_*A*_; second stage: *s*_*B*_ and *s*_*C*_), all with two available actions (*a*_*A*_ and *a*_*B*_). The model consists of model-based and model-free strategies that both learn a function *Q*(*s*, *a*) mapping each state-action pair to its expected discounted future return. On trial *t*, the first-stage state (always *s*_*A*_) is denoted by *s*_*1*,*t*_, the second-stage state by *s*_*2*,*t*_ (*s*_*B*_ or *s*_*C*_), the first- and second-stage actions by *a*_*1*,*t*_ and *a*_*2*,*t*_, and the second-stage rewards as *r*_*1*,*t*_ (always zero, there is only reward on the second stage) and *r*_*2*,*t*_.

*Model-free strategy*. The model-free agent uses the SARSA(λ) temporal difference learning algorithm [52], which updates the value for each state-action pair (*s*, *a*) at stage *i* and trial *t* according to:

$$Q_{MF}(s,a)=Q_{MF}(s,a)+\alpha \delta_{i,t}e_{i,t}(s,a)$$

where

$$\delta_{i,t}=r_{i,t}+Q_{MF}(s_{i+1,t},a_{i+1,t})-Q_{MF}(s_{i,t},a_{i,t})$$

is the reward prediction error, α is the learning rate parameter (which determines to what degree new information is incorporated), and *e*_*i*,*t*_(*s*,*a*) is an eligibility trace set equal to 0 at the beginning of each trial and updated according to

$$e_{i,t}(s_{i,t},a_{i,t})=e_{i-1,t}(s_{i,t},a_{i,t})+1$$

before the Q-value update. The eligibilities of all state-action pairs are then decayed by λ after the update.

We now describe how these learning rules apply specifically to the two-step task. The reward prediction error is different for the first two levels of the task. Since *r*_*1*,*t*_ is always zero, the reward prediction error at the first stage is driven by the value of the selected second-stage action *Q*_*MF*_(*s*_2,*t*_,*a*_2,*t*_):

$$\delta_{1,t}=Q_{MF}(s_{2,t},a_{2,t})-Q_{MF}(s_{1,t},a_{1,t})$$

Since there is no third stage, the second-stage prediction error is driven by the reward *r*_*2*,*t*_:

$$\delta_{2,t}=r_{2,t}-Q_{MF}(s_{2,t},a_{2,t})$$

Both the first- and second-stage values are updated at the second stage, with the first-stage values receiving a prediction error down-weighted by the eligibility trace decay, λ. Thus, when λ = 0, only the values of the current state get updated.

*Model-based strategy*. The model-based algorithm works by learning a transition function that maps the first-stage state-action pairs to a probability distribution over the subsequent states, and then combining this function with the second-level model-free values (i.e., the immediate reward predictions) to compute cumulative state-action values by iterative expectation. In other words, the agent first decides which first-stage action leads to which second-stage state, and then learns the reward values for the second-stage actions.

At the second stage, the learning of the immediate rewards is equivalent to the model-free learning, since those Q-values are simply an estimate of the immediate reward *r*_*2*,*t*_. As we showed above, the SARSA learning rule reduces to a delta-rule for predicting the immediate reward. This means that the two approaches coincide at the second stage, and so we set *Q*_*MB*_ = *Q*_*MF*_ at this level.

The model-based values are defined in terms of Bellman’s equation [37], which specifies the expected values of each first-stage action using the transition structure *P* (assumed to be fully known to the agent):

$$Q_{MB}(s_{A},a_{j})=P(s_{B}|s_{A},a_{j})\underset{a\in \{a_{A},a_{B}\}}{max}Q_{MF}(s_{B},a)+P(s_{C}|s_{A},a_{j})\underset{a\in \{a_{A},a_{B}\}}{max}Q_{MF}(s_{C},a)$$

where we have assumed these are recomputed at each trial from the current estimates of the transition probabilities and second-stage reward values.

*Decision rule*. To connect the values to choices, the Q-values are mixed according to a weighting parameter *w*:

$$Q_{net}(s_{A},a_{j})=wQ_{MB}(s_{A},a_{j})+(1-w)Q_{MF}(s_{A},a_{j}).$$

Again, at the second stage the decision is made using only the model-free values. We used the softmax rule to translate these Q-values to actions. This rule computes the probability for an action, reflecting the combination of the model-based and model-free action values weighted by an inverse temperature parameter. At both states, the probability of choosing action *a* on trial *t* is computed as

$$P(a_{i,t}=a|s_{i,t})=\frac{exp(\beta Q_{net}(s_{i,t},a))}{\sum_{a\prime }exp(\beta Q_{net}(s_{i,t},a\prime ))}$$

where the inverse temperature *β* determines the randomness of the choice. Specifically, when *β* → ∞ the probability of the action with the highest expected value tends to 1, whereas for *β* → 0 the probabilities over actions becomes uniform.

#### Simulation of the accuracy-demand trade-off

In order to test whether the Daw two-step task embodies a trade-off between goal-directed behavior and reward, we estimated the relationship between control (model based vs. model free) and reward by Monte Carlo simulation.

**Fig 3:**
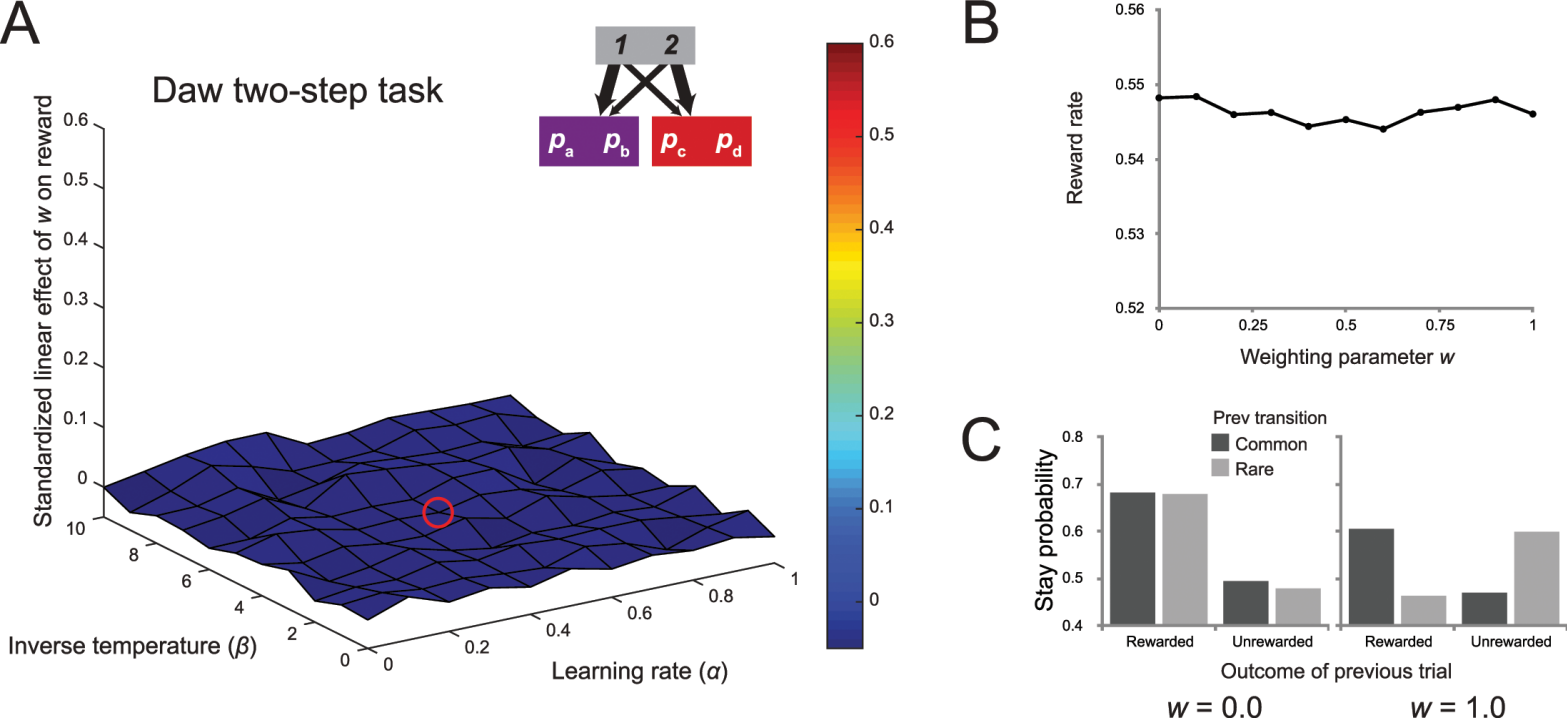
Results of simulation of accuracy-demand trade-off in the Daw two-step task. (A) Surface plot of the standardized linear effect of the weighting parameter on reward rate in the original version of the two-step task. Each point reflects the average of 1000 simulations of a dual-system reinforcement-learning model of behavior of this task with different sets of drifting reward probabilities, as a function of the learning rate and inverse temperature of the agents. The red circle shows the median fit. Importantly, across the entire range of parameters, the task does not embody a trade-off between habit and reward. (B) An example of the average relationship between the weighting parameter and reward rate with inverse temperature = 5.0 and α = 0.5 (mirroring the median fits reported by Daw and colleagues [8]) across 1000 simulations. (C) The probabilities of repeating the first-stage action as a function of the previous reward and transition for a purely model-free agent and purely model-based agent.

For each simulation, we generated a new set of four series of independently drifting reward probabilities across 201 trials according to a Gaussian random walk (mean = 0, *σ* = 0.025, the same parameters used by Daw and colleagues [8]) with reflecting boundaries at 0.25 and 0.75 (also used by Daw and colleagues). Then we simulated performance on the task for 11 different values of the weighting parameter *w*, ranging from 0 to 1, the inverse temperature, ranging from 0 to 10, and the learning rate, ranging from 0 to 10. For each of these, we recorded the reward rate obtained. Next, we ran a linear regression for each combination of inverse temperature and learning rate, predicting the reward rate from the size of the weighting parameter. Note that the data points in each linear regression were generated using the same set of drifting rewards, ensuring that any effect was due to the changes in the weighting parameter and not to random variation across the reward distributions themselves. The eligibility trace parameter was fixed at a value that corresponded approximately with previous reports of this task, *λ* = 0.5, but we found qualitatively identical results across all simulations when we fixed *λ* at 0 or 1 (see Supporting Information). We repeated this process 1000 times, computing a surface of regression coefficients across a range of reinforcement learning parameters, and then then averaged across these surfaces. The results of this analysis can be seen in the surface plot in Fig 3A, where we have plotted the average standardized linear effect of the weighting parameter as a function of the learning rate and inverse temperature (the median fit is indicated by the red circle on the surface in Fig 3A).

The striking feature of this surface map is that the regression coefficients are uniformly close to zero, indicating that none of the parameterizations yielded a linear relationship between model-based control and reward rate. Fig 3B provides a more fine-grained picture of this relationship for a specific parameterization that follows the median fits reported by Daw and colleagues [8]. Note that even though there is no significant relationship between reward and model-based control, this does not undermine the usefulness of the task for measuring the relative balance of model-based and model-free strategies (see Fig 3C). What we can conclude is that this balance does not embody a trade-off between accuracy and demand.

### Simulations of related tasks

Since its conception, the design of the Daw two-step task has been used in many similar sequential decision making tasks. Given the surprising absence of the accuracy-demand trade-off in the original task, it is important to investigate whether related versions of this paradigm are subject to the same shortcoming.

#### Dezfouli and Balleine two-step task

**Fig 4:**
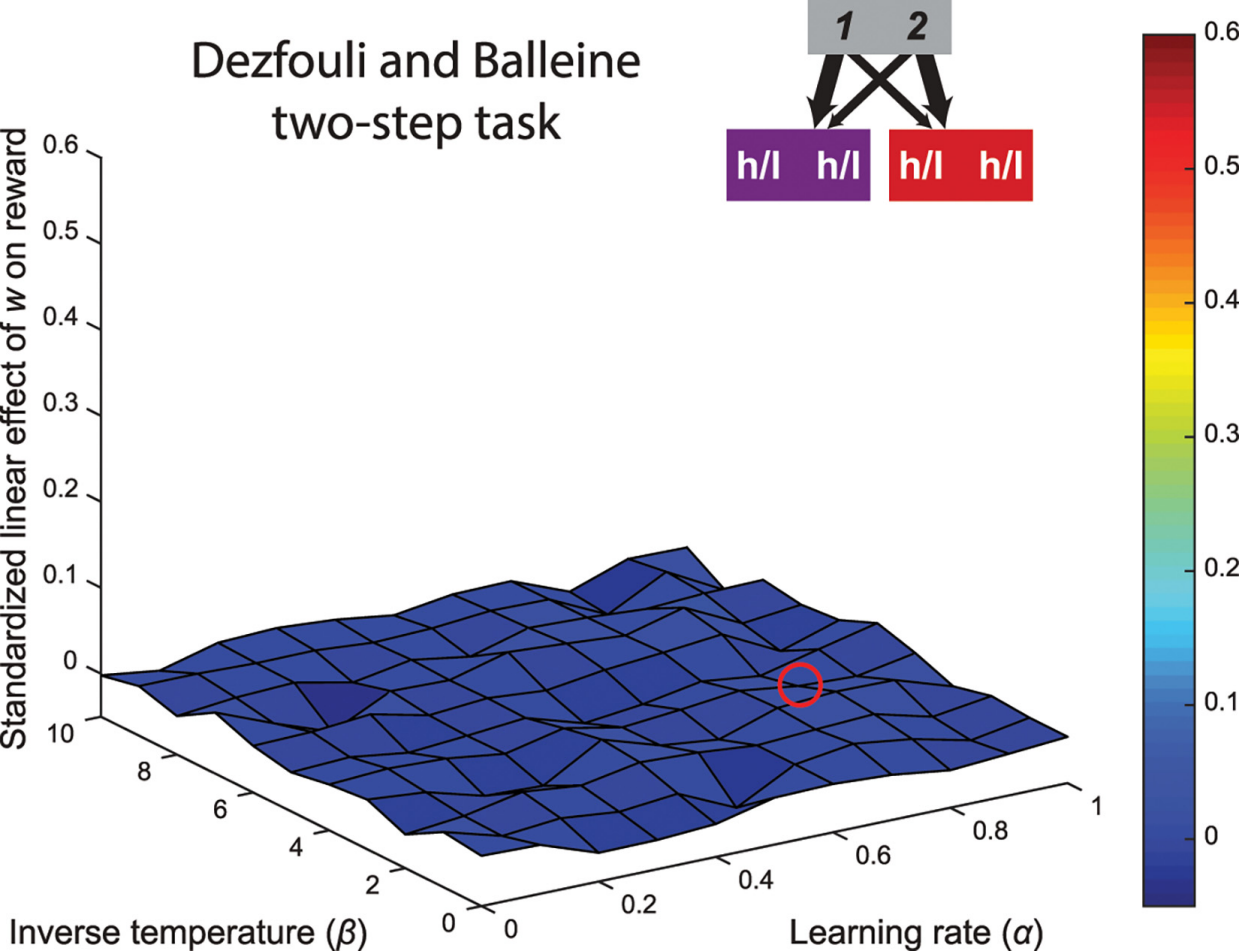
Surface plot of the linear relationship between the weighting parameter and reward rate in the Dezfouli and Balleine version of the two-step task. The red circle shows the median fit. Similar to the Daw variant, this task does not capture a trade-off between accuracy and demand across all tested parameterizations.

In one of these variants, developed by Dezfouli and Balleine [25, 26], participants also navigate from one first-level stage to two second-level stages, utilizing the same common and rare transition structure as in the Daw two-step task, but the reward probabilities are implemented in different fashion. Instead, choices at the second stage have either a high probability (0.7) or a low probability (0.2) of winning, and on every trial the probability of each second-level action changes randomly to either the high or low probability with a small probability (0.2). This task dissociates model-based and model-free control in a manner similar to the Daw two-step task. We performed the same analysis as reported in the previous section for this task, and the results were strikingly similar (see Fig 4). As before, across the entire range of reinforcement learning parameters, the task did not exhibit a trade-off between demand and accuracy, evidence by the uniformly flat regression coefficients (the median fit is represented by the red circle on the surface of Fig 4).

#### Doll two-step task

**Fig 5:**
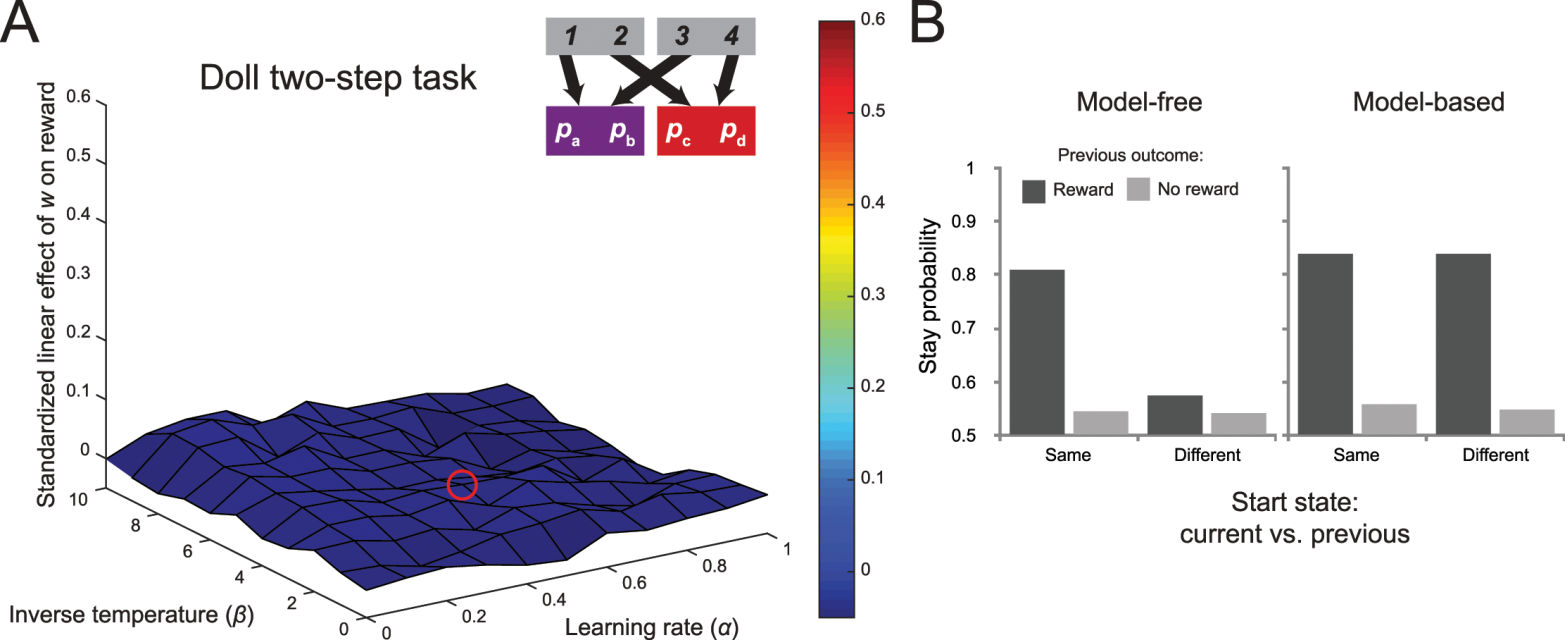
Results of simulation of the Doll two-step task. (A) Surface plot of the linear relationship between the weighting parameter and reward rate in the Doll version of the two-step task. The red circle shows the median fit. Similar to the Daw variant, this task does not capture a trade-off between accuracy and demand across all tested parameterizations, except for a slightly elevated region of parameter space with high inverse temperature and low learning rate. (B) Behavioral predictions in this task. The model-free system learns separate values for each action in each state, so outcomes only affect choices in the same start state. Our simulation of model-free behavior revealed elevated likelihood of staying after a reward from the other state, since this means there is a current high-probability option that the model-free system has been learning about after transitioning there from both start states. The model-based system (on the right) treats start states as equivalent, since they both afford the same transitions, so choices are not affected by whether the previous start state was the same or different.

A second variant, reported by Doll and colleagues [15], uses two first-stage states, but the choices at these states transition deterministically to one of the two second-stage states (Fig 5A). At the second stage the choices have chance of producing reward, with probabilities slowly changing over the course of the experiment according to the same Gaussian walk (mean = 0, *σ* = 0.025) as in the original task with reflecting bounds at 0.25 and 0.75.

The dissociation between habit and planning in this task follows a different logic. Here, it is assumed that only model-based learners use the implicit equivalence between the two first-stage states, and can generalize knowledge across them. Therefore, for a model-based learner, outcomes at the second level should equally affect first-stage preferences on the next trial, regardless whether this trial starts with the same state as the previous trial or a different one. For model-free agents, however, rewards that are received following one start state should not affect subsequent choices from the other start state. According to Doll and colleagues [15], this results in a clear dissociation in staying behavior between these two strategies (Fig 5B): The model-based learner shows increased likelihood to stay with the choice made on the previous trial when this led to a reward, regardless of whether the start-state is the same as or different from the start state on the previous trial. (Note that here the term ‘staying’ is used to refer to taking the action that leads to the same previous second-stage state, and not to describe a repetition of the same first-stage action.) The model-free learner, on the other hand, is argued to only show increased likelihood to repeat a choice after a reward when the current start state is the same as that on the previous trial. Behavioral performance on this task is consistent with these predictions, and compared to the original two-step task, seems to reflect a mixture of model-based and model-free strategies [15].

However, our simulations revealed that the behavioral profile for the model-free learner also showed a slightly elevated likelihood to stay with the previous choice after a reward if the start state was different (Fig 5B). On the first glance, this may seem surprising because the model-free system does not have access to the second-stage action values. Note, though, that this system builds up first-stage action values based on the previous reward history. If one particular action has a high chance of producing reward for an extended period of trials, then the model-free system will learn to choose actions in order to transition to the relevant state, resulting in increased stay behavior. It is important to note that in this task the effect is small, and only becomes reliable with large sample sizes (we simulated 1000 reinforcement-learning agents). Regardless, this observation makes the interpretation of raw stay probabilities less clean, since an elevated likelihood to repeat the previous action after a reward with a different start-state is not simply attributable to a contribution of the model-based system. This places extra importance on fitting computational models to behavior in this task, since these incorporate reward histories over the entire experiment, which are omitted when analyzing raw choice behavior on single trials. (This point becomes crucial in subsequent experiments described below.)

In order to assess whether the Doll two-step task embodied a trade-off between accuracy and demand, we again estimated the relationship between the weighting parameter and reward rate. This analysis (depicted in Fig 5A) suggested that this relationship was largely similar to the results reported above, and very close to zero for large portions of the parameter range. However, in comparison with the Daw and Dezfouli versions, the Doll task showed increased sensitivity in a parameter space with relatively high inverse temperatures (low randomness) and small learning rates. It is important that even in this elevated part of the coefficient surface the hypothetical effect is small, and that the participants’ parameter fits did not fall in this parameter range (the mean fit is indicated by the red circle on the surface in Fig 5A).

### Factors contributing to the absence of the trade-off

Despite the substantial differences between these variants of the two-step task, we found that none of them encompasses a motivational trade-off between planning and reward. This observation naturally raises a question: Why does planning not produce an increased reward rate in this task? What characteristics of the paradigm distort the accuracy-demand trade-off?

We investigate five potential explanations. These are not mutually exclusive; rather, they may have a cumulative effect, and they may also interact with each other. First, we show that the sets of drifting reward probabilities that are most often employed are marked by relatively low distinguishability. Second, we show that the rate of change in this paradigm is slow and does not require fast online (model-based) flexibility. Third, we show that the rare transitions in the Daw two-step task diminish the reward-maximizing effect of a model-based choice. Fourth, we show that the presence of the choice at the second stage decreases the importance of the choice at the first stage, which is the only phase where the model-based system has an influence. Fifth, we show that the stochastic reward observations in this task do not carry enough information about the value of the associated stimuli. We use simulations of performance on novel tasks to demonstrate these five points and, as a result, develop a novel paradigm that embodies an accuracy-demand trade-off.

#### 1. Distinguishability of second-stage probabilities

**Fig 6:**
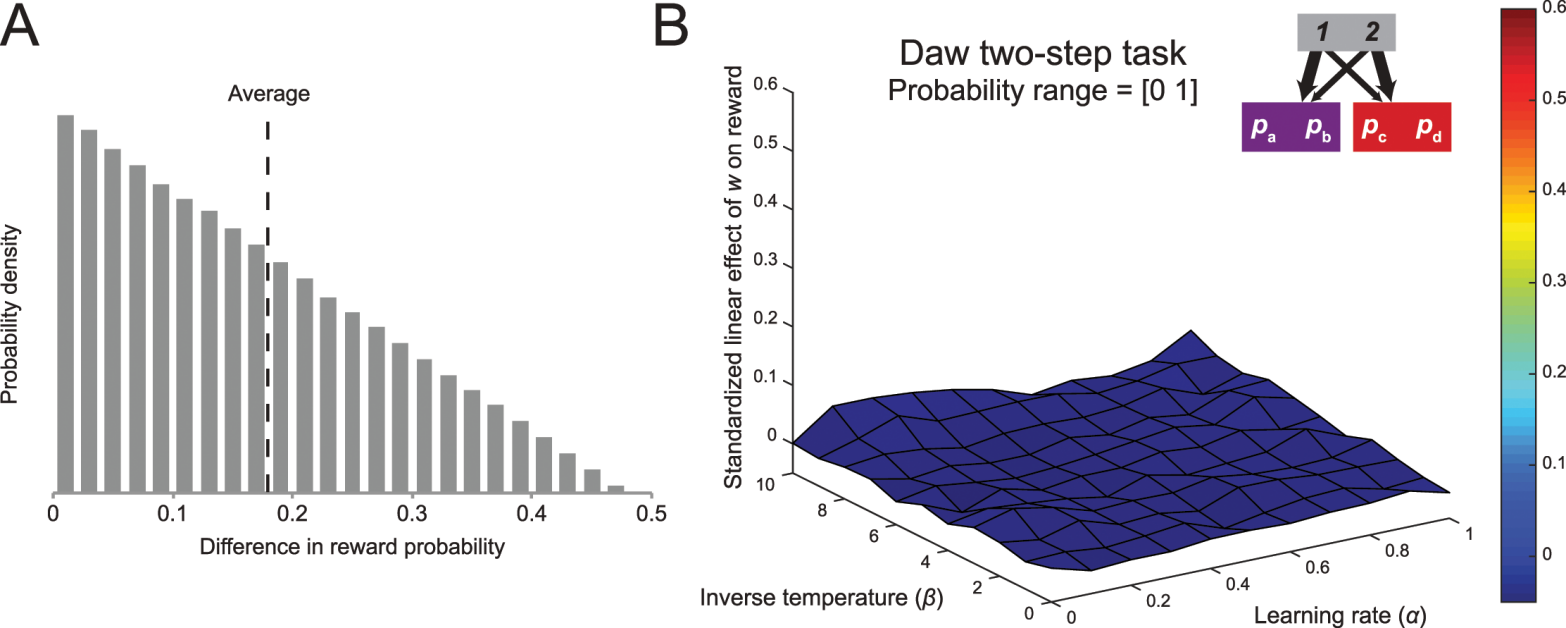
The influence of the range of reward probabilities. (A) Distribution of differences in reward probabilities between the actions of each trial. (B) Increasing the range of probabilities increases the average linear effect between model-based control and reward for a parameter space associated with high inverse temperatures and relatively low learning rate. Average parameter fits in the original report do not lie within this region of increased sensitivity to the accuracy-demand trade-off.

In the two-step task, the difference between model-based and model-free strategies only carries consequences for the first stage, since the second stage values are identical for both strategies. Therefore, the finding that model-based control is not associated with an increased reward rate suggests that the first-stage choices the agent makes do not carry importance, for example, because the reward outcomes at the second stage are too similar. In the original version of the two-step task, the reward probabilities have a lower bound of 0.25 and an upper bound of 0.75. This feature results in a distribution of differences between reward probabilities that is heavily skewed left (Fig 6A). The mean value of this distribution approaches 1/6 (1/3 of the range of 0.5), suggesting that most choices in this task only carry modest consequences, because the associated values have relatively low distinguishability.

One straightforward way to increase the differences between the second-stage options is to maximize the range of reward probabilities by setting the lower bound to 0 and the upper bound to 1 (e.g., [20]). This shifts the mean difference in reward probability between options to 1/3, doubling the consequences of each choice in terms of reward maximization. As can be seen in Fig 6B, this change in the paradigm slightly increases the degree to which behavior in this task reflects an accuracy-demand trade-off (Simulation 1). The regression coefficients of the relationship between reward and *w* are slightly elevated compared to those of the Daw two-step task (compare with Fig 1), especially in the part of parameter space with high inverse temperature and low learning rate, suggesting that low distinguishability between the options is a factor contributing to the absence of the trade-off. However, the increase in the effect is fairly small, and the parameter space at which the highest increase is observed does not correspond with the average parameter fits reported in the literature.

#### 2. Increased drift rate

It is also possible that the changes over time in the second-stage reward probabilities, depicted in Fig 1B, contribute to the absence of the accuracy-demand trade-off in the Daw two-step task. For example, these changes might be too slow, such that model-free learning can adapt to these values at a rate that is proportional to the rate of change over time [53]. Another possibility is that these changes happen too fast, such that the model-based system never accurately reflects the values of the second-stage actions.

In order to explore the effect of the drift rate (i.e., the standard deviation of the Gaussian noise that determines the random walks of the reward distributions) on the accuracy-demand trade-off, we performed simulations of the generative reinforcement learning model with inverse temperature parameter *β* = 5 and learning rate parameter *α* = 0.5, mirroring the median fits reported by Daw and colleagues [8]. For each of these, we generated a new set of four series of independently drifting reward probabilities across 201 trials according to Gaussian random walks. We simulated performance on the task for 11 values of the weighting parameter, ranging from 0 to 1, and ran a linear regression predicting the reward rate from the size of the weighting parameter. Again, this method ensured that the data points in each linear regression were generated using the same set of drifting reward probabilities, ensuring that any effect was due to the changes in the weighting parameter. This analysis was performed for 7 different drift rate values, ranging from 0 to 0.5, using the narrow range of reward probabilities used in the original report [8] and the broader range described above. The slopes we report will be the average across a large number of iterations (10,000), since we are estimating very subtle effects, especially in the narrow range condition.

**Fig 7:**
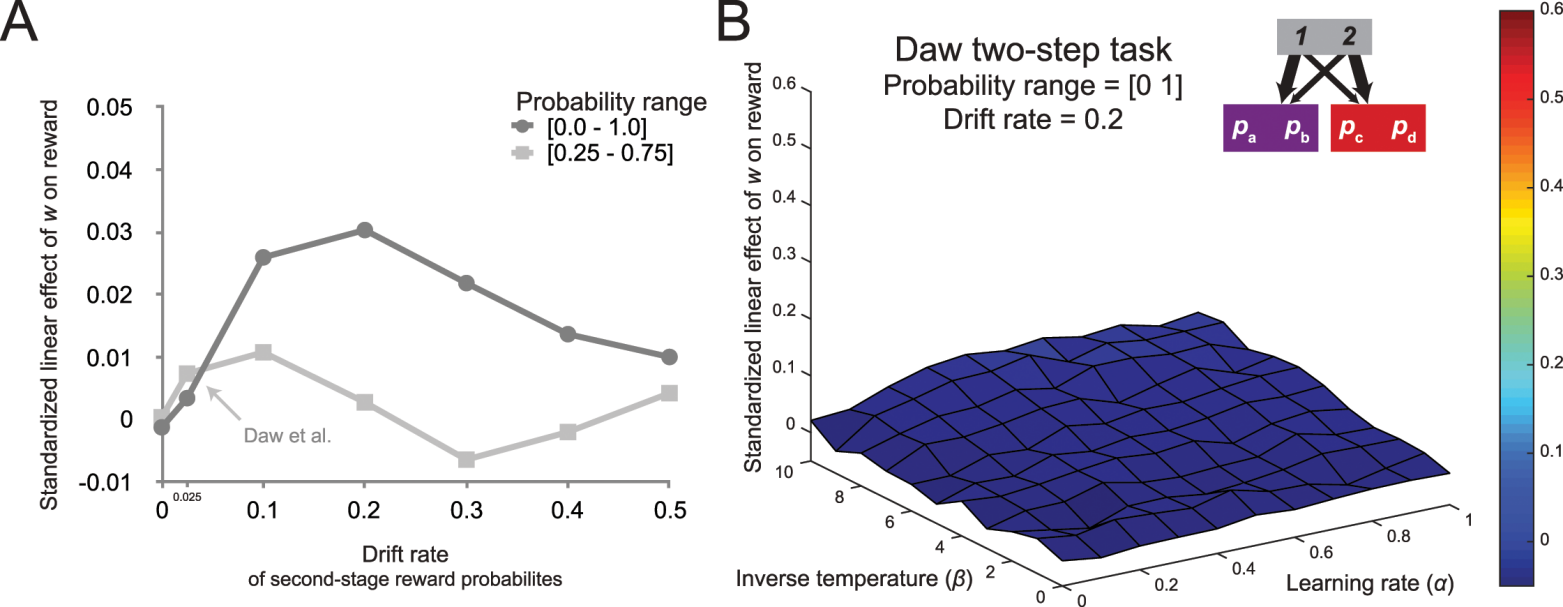
The influence of the drift rate. (A) The effect of the size of the drift rate on the relationship between model-based control and reward, for two-step tasks with a narrow and a broad reward probability range. (B) Increasing the range of probabilities and the drift substantially increases the average linear effect between model-based control and reward when the inverse temperature is high.

The results, depicted in Fig 7A, suggest that the drift rate of the Gaussian random walk affects the strength of the accuracy-demand trade-off in the task. Specifically, for the broader range of parameterizations, the strongest relationship was observed for a drift rate of 0.2, and for the parameterization range of the original report the maximum effect occurred with a drift rate of 0.1. Consistent with the section above, both the strength of the relationship between reward and planning, as well as the effect of the drift rate on this relationship was stronger for the task with the broader reward probability range. Note however, that for both probability ranges large drift rates negatively impact the relationship, presumably because for these values there is no learnable stability in the terminal state reward probabilities. We confirmed that this general pattern between the accuracy-demand trade-off, the drift rate, and the range of the reward probabilities occurs not only at the parameterization that most closely matched the estimates of the original report, but also across a broader parameter space (see Supporting Information). Fig 7B depicts the surface map of average regression coefficients for the effect between model-based control and reward rate for a task with the wider reward probability range and a drift rate of 0.2 (Simulation 2). These changes to the task substantially increase the strength of the accuracy-demand relationship, especially for agents with a high inverse temperature. However, as before, the increase in the strength of this relationship primarily occurred in regions of parameter space that do not correspond with the fits reported in studies that employ the Daw two-step task [8, 41], and had a very weak effect size.

These analyses show that the rate of change of the reward probabilities in the original Daw two-step task is too slow to promote model-based planning. The relationship between reward and model-based control becomes stronger when the drift rate of the Gaussian random walk governing the reward probabilities is moderately increased, and this effect is especially pronounced when these probabilities are more dissociable. However, even though these two factors contribute substantially to the absence of the accuracy-demand trade-off in the Daw two-step task, we found that a task that adjusted for their shortcomings only obtained a modest trade-off between reward and goal-directed control.

#### 3. Deterministic transition structure

Because the Daw two-step task employs rare transitions, model-based choices at the first stage do not always lead to the state that the goal-directed system selected. This feature of the task might lead to a weakening of the relationship between model-based control and reward rate. The task structure employed by Doll and colleagues [15], discussed in the previous section (Fig 5A), avoids this issue by implementing deterministic transitions, such that model-based choices always lead to the desired second-stage state. Indeed, the simulation analysis for this task revealed that a certain range of the simulated parameter space showed an increased relationship between model-based control and reward rate. However, for this analysis the set of drifting rewards still suffered from the factors discussed above–relatively low distinguishability of the second-stage options and a suboptimal drift rate.

**Fig 8:**
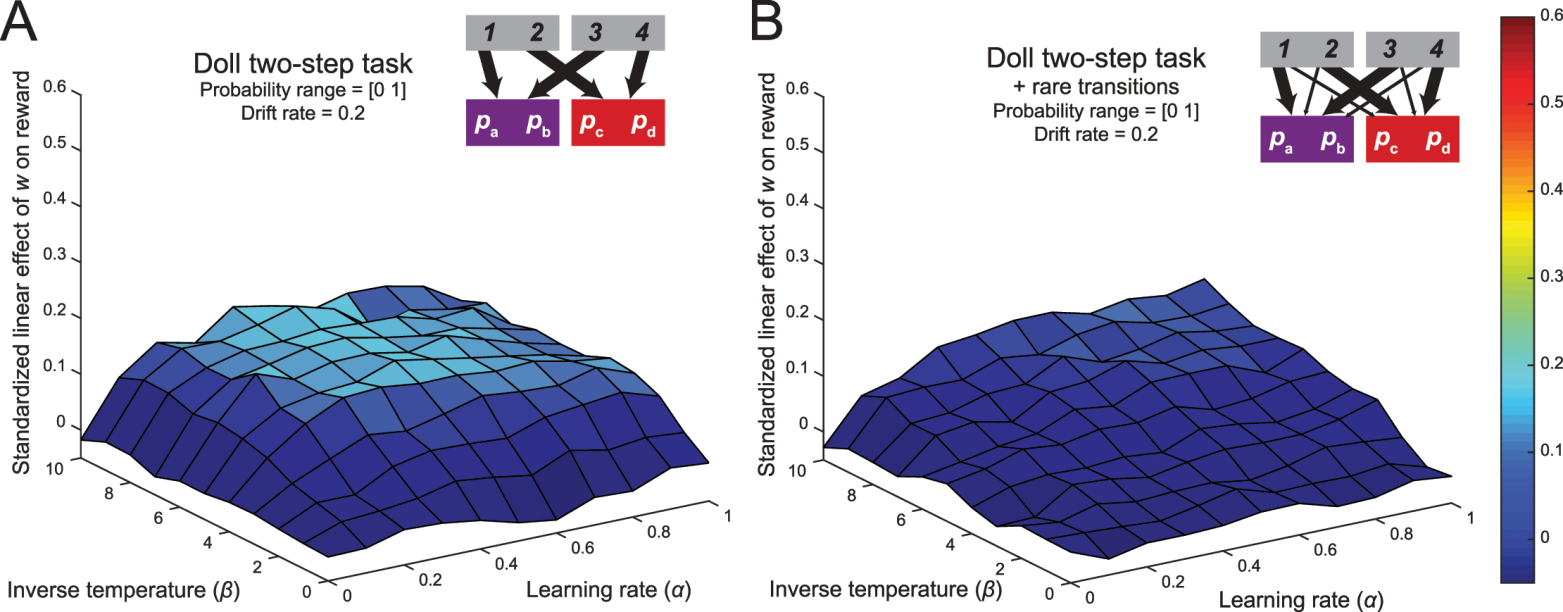
The influence of a deterministic task structure. (A) Because of the deterministic transitions, model-based choices in the Doll two-step task always result in the desired state outcome. Combined with increased distinguishability and increased drift rate in the reward probabilities, this task results in a substantial increase in the relationship between planning and reward. (B) When this task structure is adapted to include stochastic transitions, the relationship between planning and reward is significantly reduced, indicating an important contribution of the rare transitions in diminishing the accuracy-demand trade-off in the original paradigm.

To assess the influence of the deterministic task structure, we simulated performance on the Doll version of the two-step task with sets of reward probabilities with the wider range and increased drift rate (a bounded Gaussian random walk with *μ* = 0, *σ* = 0.2 on a range from 0 to 1 with reflecting bounds). The resulting surface map (Simulation 3a; Fig 8A) showed that, consistent with our predictions, across the entire parameter space the relationship between control and reward was stronger when compared to the Daw two-task with improved sets of reward probabilities in the previous section.

Even though this result is consistent with the assumption that model-based choices in the Daw two-step task lead to the desired state less often than in the deterministic version of the two-step task, it is equally possible that the second task shows an increased accuracy-demand trade-off because it introduces the possibility of generalization across actions, and not because of the elimination of the rare transitions. To disentangle these two possibilities, we simulated reinforcement-learning performance on a hybrid task with two starting states but with rare transitions (Simulation 3b; Fig 8B). The regression coefficients in the resulting surface map were substantially lower than in the deterministic variant of the task, and was comparable to that of the Daw two-step task with broader probability range and a higher drift rate. This indicates a critical role for the rare transitions in diminishing the accuracy-demand trade-off.

#### 4. One choice in second stage

As noted above, model-based and model-free strategies make divergent choices only at the first stage of the multi-step paradigms we have considered so far; at the second stage, both strategies perform a biased selection weighted towards the reward-maximizing option. Thus, the advantage of model-based control over model-free control is approximately bounded by the difference between the maximum value of all actions available in one second-stage state and the maximum value of all actions available in the other second-stage state. Intuitively, as the number of actions available within each second-stage state grows, this difference will shrink, because both second-stage states will likely contain some action close to the maximum possible reward value (i.e., a reward probability of 1). Conversely, the difference between the maximum value actions available in each second-stage state will be greatest when only a single action is available in each state. This design should favor the largest possible difference in the rate of return between model-based and model-free strategies.

To quantify this, we generated 10,000 sets of reward probabilities in this task (according to a Gaussian random walk with reflecting bounds at 0 and 1 and *σ* = 0.2). The average difference between *any two* reward probabilities within a state was equal to 0.33, whereas the average difference between the *maximal* reward probabilities of the two states was 0.27.

Since the model-based system only contributes to the first-stage decision, we simulated performance of the reinforcement-learning model in a deterministic two-step task in which the second-stage states do not contain a choice between two actions. In this task, the average difference in reward probabilities that the model-based system uses to make a choice at the first stage is 33%, an increase in comparison to the task that implements a binary choice at the second stage states.

**Fig 9:**
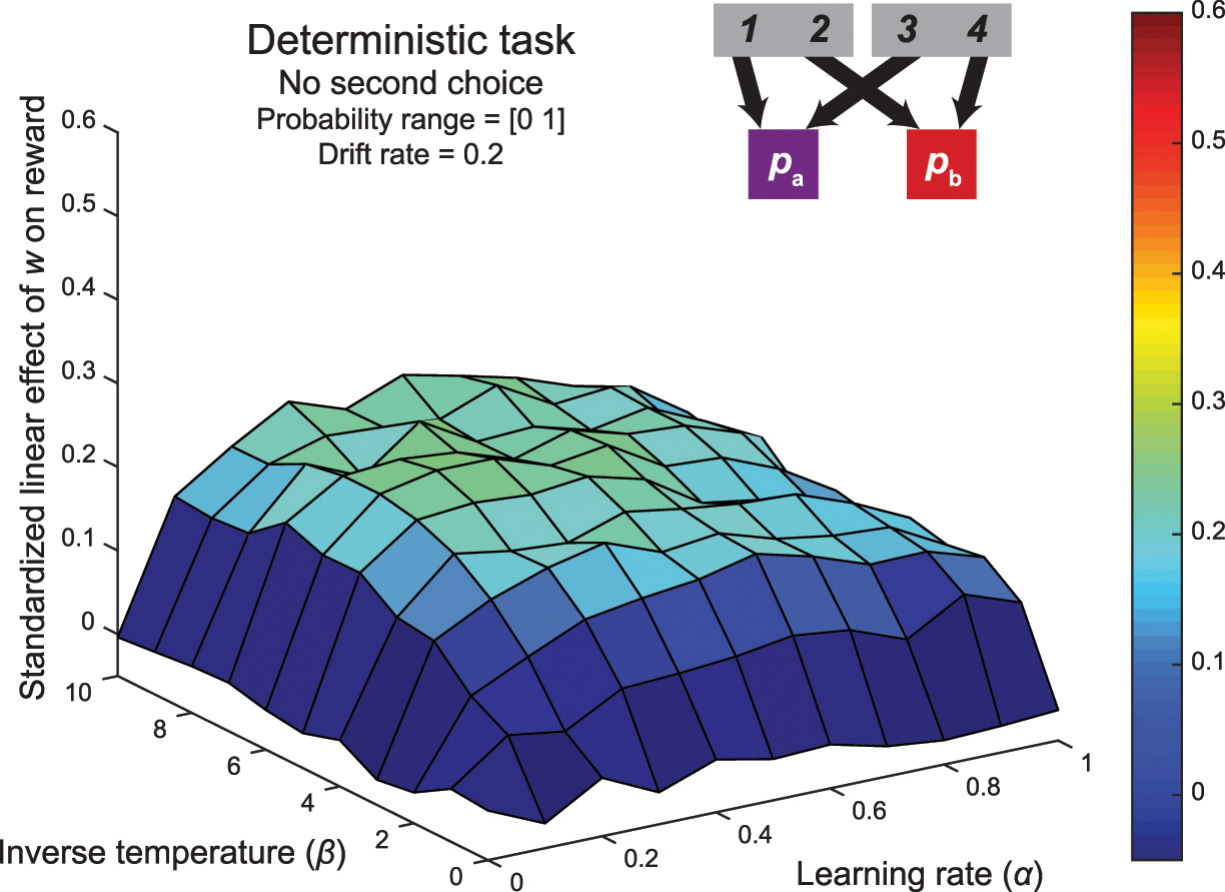
The influence of reducing the number of second-stage action. Because of the deterministic transitions, model-based choices in the Doll two-step task always result in the desired state outcome. Combined with increased distinguishability and increased drift rate in the reward probabilities, this task results in a substantial increase in the relationship between planning and reward.

To assess whether this change to the task resulted in a stronger accuracy-demand trade-off, we simulated performance on this task and estimated the strength of the relationship between the weighting parameter and reward rate, across the same range of reinforcement-learning parameters (Simulation 4; Fig 9). This analysis revealed that the elimination of the choices at the second stage indeed strengthened the relationship between *w* and reward in comparison to the deterministic task with second-stage choice (Fig 8A), because the larger difference between ‘maximal’ reward probabilities between the two second-stage states increased the importance of the model-based contribution to the first-stage choice.

#### 5. Informativeness of an observation

In order to determine the value of an action in the two-step task, the stochastic nature of the task requires participants to sample the same action repeatedly and integrate their observations. In other words, since each outcome is either a win or a loss, the information contained in one observation is fairly limited. Here, we will test whether the high amount of ambiguity associated with each observation contributes to the absence of the accuracy-demand trade-off in the two-step task. One way to increase the informativeness of an outcome observation is to replace the drifting reward probabilities at the second stage with drifting scalar rewards, so that the payoff of each action is exactly identical to its value [42]. This elimination of uncertainty increases the information obtained from each outcome observation, and thus may lead to a strengthened relationship between model-based control and reward.

**Fig 10:**
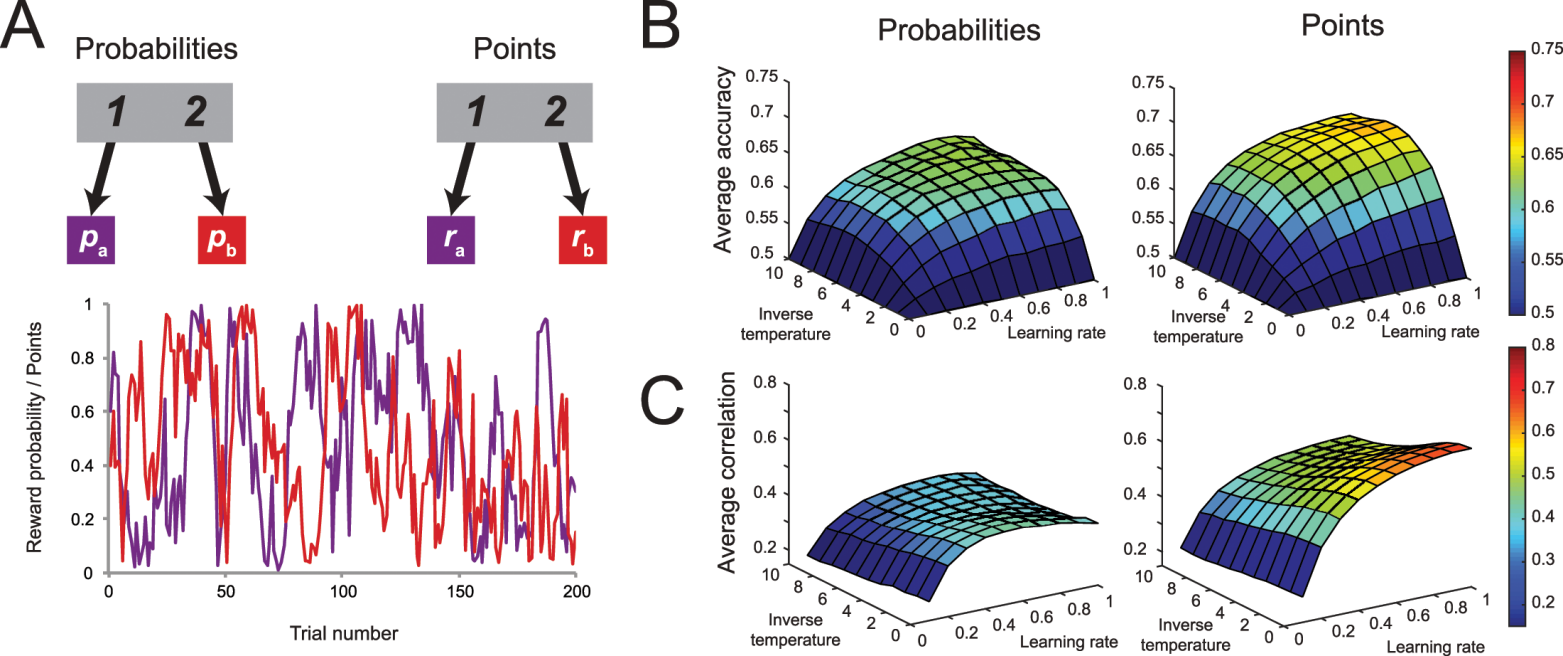
The influence of the type of reward distribution (points vs probabilities) on choice accuracy. (A) We ran simulations of RL agents on two different two-armed bandit tasks. For one, the reward distributions indicate the reward probability associated with each action. The other task does not include binomial noise, but instead the actions pay off rewards that are directly proportional to its value in the reward distribution. (B) Agents show greater accuracy in choosing the highest-value action on the task the task where the two-armed bandit pays off points instead of affording a probability to win a reward, especially when both the inverse temperature and learning rate were high. (C) The Q-values of each action shows stronger correlations with their objective reward value in the task where the two-armed bandit payed off points instead of affording a probability to win a reward.

In order to test whether the reward distributions for the second-stage actions would improve the information obtained from each observation, we performed a series of simulations for two simple reinforcement learning tasks (Fig 10A). Both tasks involved a repeated decision between task options, but the nature of the reward for these two options was different. For one task, these actions were associated with a probability of a reward, which independently drifted across the session according to a Gaussian random walk (*μ* = 0, *σ* = 0.2) on a range from 0 to 1 with reflecting bounds. For the other task, the same series of drifting probabilities were treated as series of drifting rewards. Specifically, this meant that if an action afforded a 74% probability for a reward in the first task, the same action in the second task would lead to a payoff of 0.74 points. As before, we performed reinforcement learning simulations (*λ* = 0.5) on these two tasks across a range of inverse temperatures and learning rates. Because we used the same sets of drifting values as probabilities and payoff (i.e., their expected values are the same), any difference in performance between the two tasks is a function of the increased amount of information available in the payoff condition.

We first compared the model’s performance on these two tasks by computing the accuracy of its choices, i.e., how often it selected the action with the highest reward probability or reward payoff. Fig 10B displays the average accuracy for each task across the range of inverse temperatures and learning rates. For both tasks, the model showed increased accuracy for higher learning rates and inverse temperatures. That is, agents with less randomness in choice and greater incorporation of new information were more likely to pick the option with the objectively higher chance to win or reward payoff. Consistent with our prediction, this effect was larger in the task with reward payoffs compared to the task with reward probabilities across virtually the entire simulated parameter space. This suggests that the observation of reward outcome in the payoff condition was more informative, leading to overall better performance in the simple two-alternative choice task.

As a second metric of the information contained in each outcome observation, we computed the correlation between the model’s action values and the actual payoffs in the simulations reported above. We expected that the increased precision in outcome observations in the payoff condition would lead to a tighter coupling between the Q-values of the model and the objective values as compared to the probabilities. Fig 10C depicts the results of this analysis. In both tasks, the average correlation was strongest for high learning rates, since for these agents, new information was incorporated fully, always reflecting the latest information to the largest extent. Second, the correlation was stronger when the inverse temperature was low, presumably because agents with high randomness in choice sample from both options. Most importantly, the correlations between action and objective values was higher in the reward payoff condition than in the probability range across the entire range of tested reinforcement learning parameters. This observation provides convergent evidence that increased resolution in the outcome observations is associated with enhanced performance in reinforcement-learning tasks.

**Fig 11:**
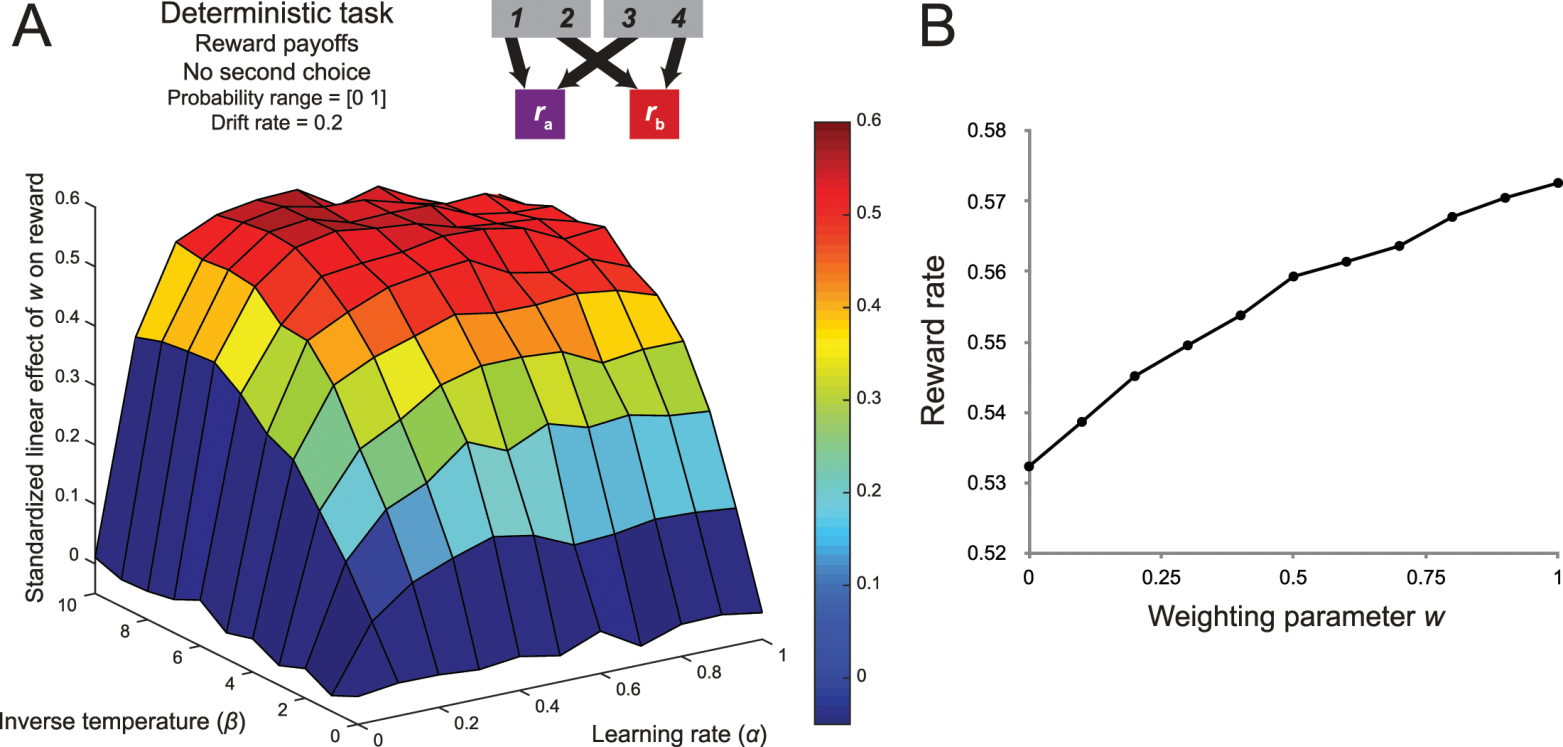
The influence of removing binomial noise from the reward distributions at the second stage. (A) The surface plot of the relationship between model-based control and reward in the novel two-step task with reward payoffs at the second stage. The inclusion of this fifth factor substantially increased the accuracy-demand trade-off in the two-step paradigm. (B) An example of the average relationship between the weighting parameter and reward rate with inverse temperature = 10 and α = 0.4.

Next, we assessed whether this increased performance in the payoff condition would result in a stronger accuracy-demand trade-off in the deterministic two-step task. We reasoned that if the agent obtained a more accurate estimation of the second-stage action values, then the model-based system would be better positioned to maximize reward. To test this prediction, we again estimated the strength of the relationship between the weighting and reward rate, across the range of reinforcement-learning parameters (Simulation 5; Fig 11). The surface map revealed a marked increase when compared to that of the task with reward probabilities. Across the entire range of inverse temperatures and learning rates, the regression coefficients of the relationship between control and reward were substantially higher in comparison to the deterministic task with reward probabilities (Fig 9). This final analysis revealed that the reward outcomes in the Daw two-step task do not carry enough information about the action’s value, leading to a decrease in accuracy for the model-based system.

#### Comparison with Akam and colleagues

In a recent study, Akam and colleagues [9] reported that the original version of the two-step task does not embody a trade-off between control and reward. They simulated performance on the task for a pure model-free and model-based agent, with independently optimized parameters to maximize reward rate, and found no difference between them, consistent with the results of our simulations. Furthermore, they proposed a new task that establishes a trade-off between control and reward. This task is similar to the original Daw version of the task, except for the elimination of the second-stage choice, a reduction of the rare transition probability (20%), and the reward probabilities in the second-stage states alternate between blocks with reward probabilities of 0.8/0.2 and blocks with probabilities of 0.2/0.8 [9]. Using optimized reinforcement learning parameters independently for model-free and model-based agents, they show that reward rate is higher for the model-based agent.

**Fig 12:**
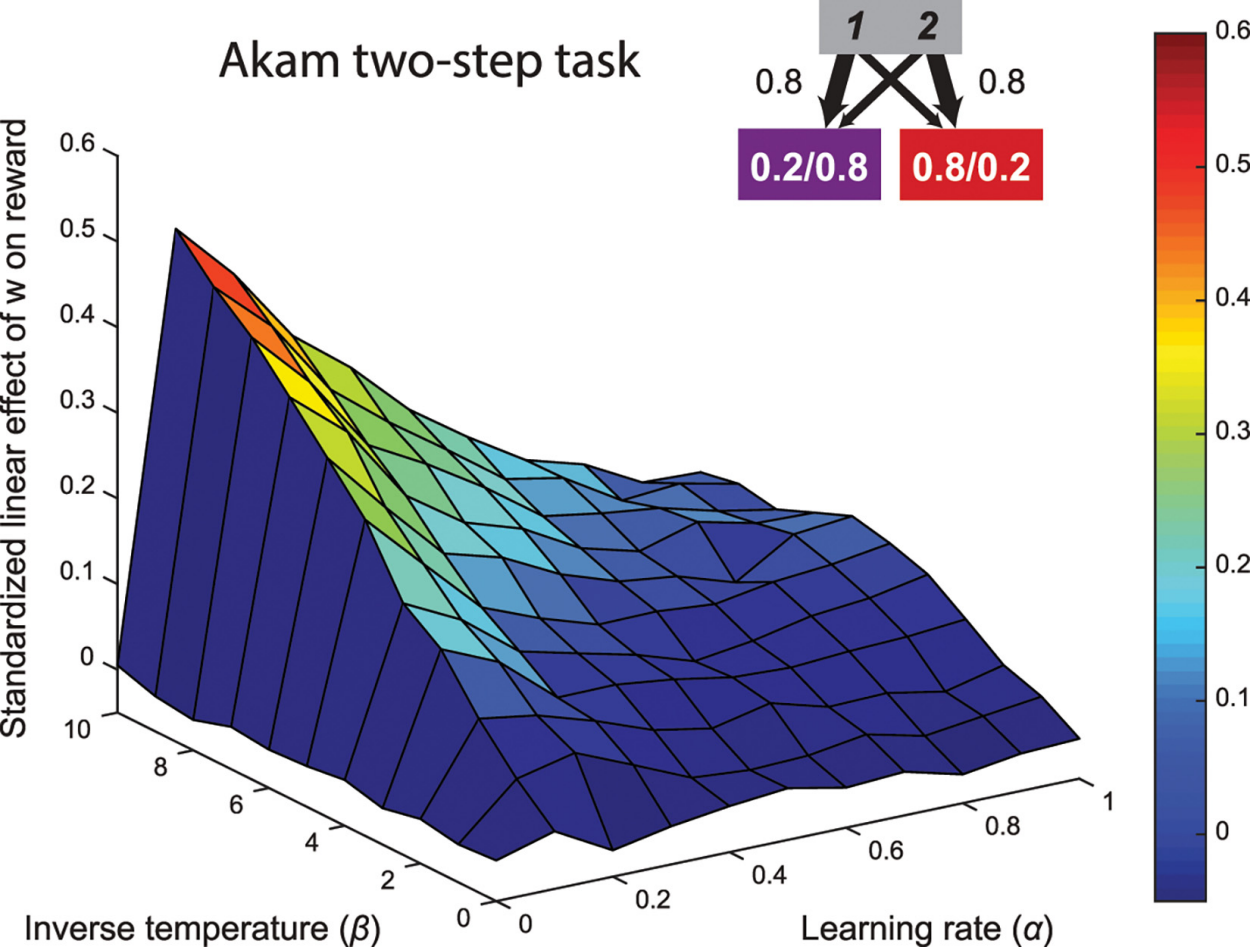
Surface plot of the relationship between model-based control and reward in the Akam and colleagues [9] version of two-step task with alternating blocks of reward probabilities at the second-stage states.

This approach—i.e., a comparison of optimal parameter settings under model-free versus model-based control—provides an important existence proof of the potential benefits of model-based control. However, their way of quantifying the accuracy-demand trade-off differs significantly from the current approach. In order get a more comprehensive overview of the accuracy-demand trade-off in the Akam two-step task, we again estimated the strength of the relationship between the weighting parameter and reward rate, across the same range of reinforcement learning parameters (Fig 12). This surface of regression coefficients shows remarkable differences compared to our novel paradigm (presented in Fig 11). Most importantly, high correlation coefficients are restricted to a selective region of parameter space with low learning rate and high inverse temperature. The strength of this relationship drops in the rest of the parameter space.

This feature of the task means that an increase in model-based control, keeping all other RL parameters fixed, is not likely to yield significantly increased total reward, because reinforcement learning parameters tend to vary widely across individuals [54]. However, it has been shown that people adapt RL parameters such as the learning rate and choice randomness to maximize reward in the environment [55], providing alleviation for this concern. A second, distinct advantage of the task we introduce here involves the possibility that humans may identify and exploit higher-level regularities in the structure of reward. Specifically, in the Akam task participants may learn to predict the alternating blocks of reward probabilities, complicating the interpretation of behavior. In contrast, in the task we introduce it is impossible to perfectly anticipate changes in our randomly changing reward distributions.

Despite these concerns, both tasks achieve an accuracy-demand trade-off, and in this respect represent a substantial improvement over the Daw two-step task. Future empirical work should compare the empirical correlations between reward and model-based control for our task and the Akam two-step task, so as to gain fuller comprehension of their respective merits.

#### Summary

We have identified several key factors that reduce the accuracy-demand trade-off in the Daw two-step task. We found that the sets of drifting reward probabilities that are most often employed in this task are marked by low distinguishability and a rate of change that is too slow to benefit from flexible online adaptation. We also showed that the rare transitions in the original task and the presence of multiple choices in the second-stage states diminished the effect of model-based decisions on reward rate. Finally, we showed that the stochastic reward observations in this task do not carry sufficient information about the value of the associated stimuli. In addition to identifying these factors, we have provided improvements to the paradigm targeting each shortcoming.

**Fig 13:**
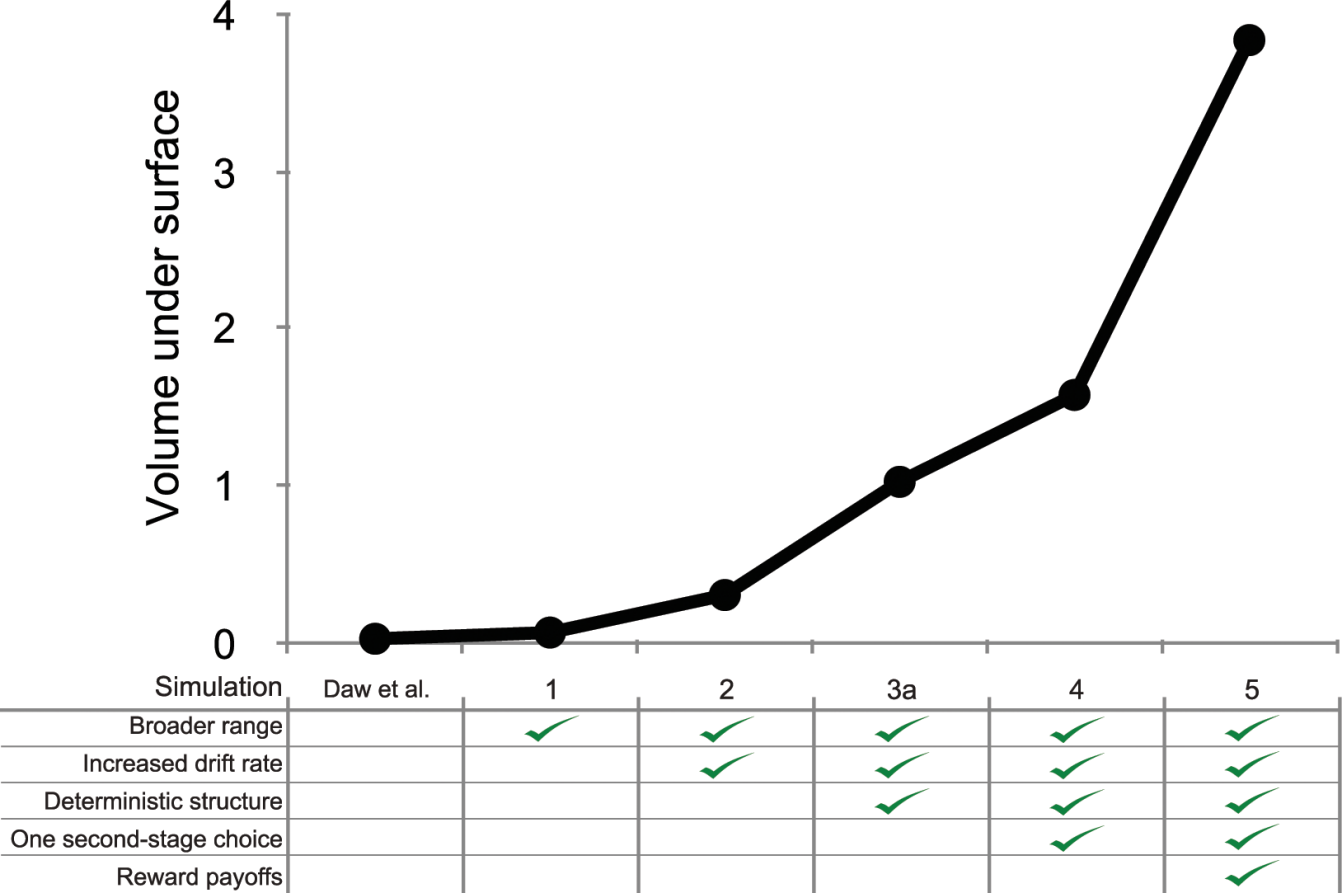
Comparison of trade-off between model-based control and reward across different paradigms. We calculated the volume under the surface of coefficients of the linear relationship between the weighting parameter and the reward rate for each of the paradigms in the section above. Across these simulations, we progressively included elements that strengthened the relationship, as summarized in this figure.

Fig 13 shows the progression in the average strength of the relationship between reward and control across these changes in the task structure, operationalized as the volume under the surface of each simulation. It reveals a progressive contribution of each change to the task, suggesting that implementing a broader range, increased drift rate, deterministic task structure, one second-stage choice, and reward payoffs all have identifiable contributions to the strength of the relationship between model-based control and reward.

Here, we have presented a progression of five factors that enhance the accuracy-demand trade-off in the two-step task. Which of these factors contributed most to the increase in this strength? Fig 13 represents the increase in this strength as additional factors are layered into the paradigm, and from this figure one might conclude that the conversion from reward probabilities to reward payoffs carried the strongest contribution. So far, however, we have confounded the contribution of each factor with the order in which they were represented. It is possible that the reward payoffs contributed a substantial amount of strength to the trade-off simply because it was the final factor introduced.

**Fig 14:**
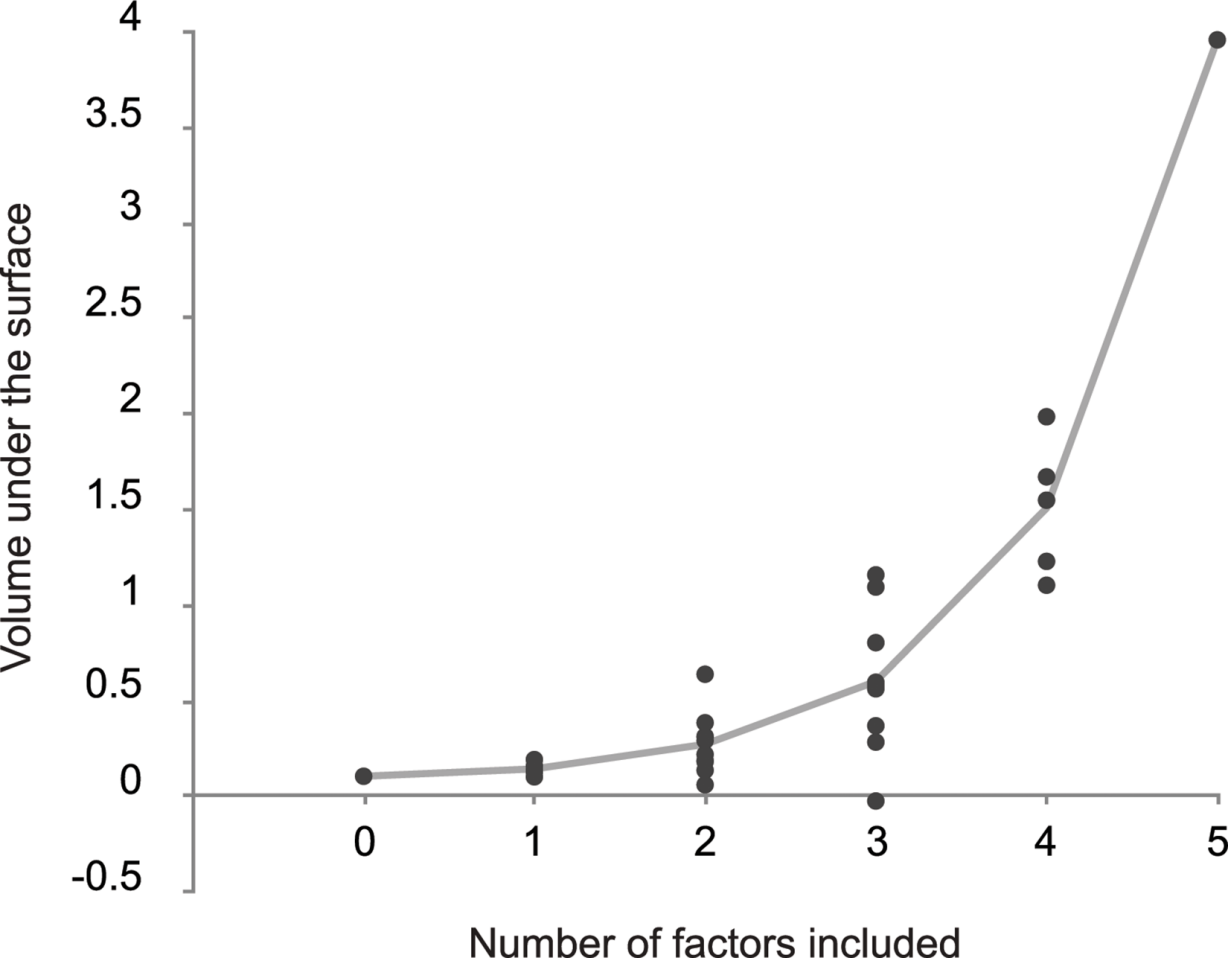
Volume under the surface for all 32 tasks generated by the 5 binary factors discussed in this paper. Each dot represents the volume under the surface of linear regression coefficients for one task, and is plotted as a function of the number of ‘beneficial’ factors that are included in each task’s design. The gray line represents the average increase in the strength of the relationship between model-based control and reward.

In order to test the effect of factor order in our analyses, we computed the surface of regression coefficients for all 32 possible combinations of our binary factors (2^5^), using the same procedure as described above (omitting the cases where β = 0, or α = 0). Next, we computed the volume under the surface as an approximation of the average strength of the relationship between model-based control and reward for each these simulations. Fig 14, depicts the volume under the surface of each simulation as a function of the number of factors that were included in the design. These results indicate that the primary cause for the strength in the final paradigm was the inclusion of all five factors, and not necessarily the contribution of one of them. To see this, compare the score of the final task with 5 included factors to the scores of the task with 4 factors. The strength of the effect in the final task was 6.9 standard deviations removed from the tasks with 4 factors, and 5.7 standard deviations from the 4-factor task with the strongest average accuracy-demand trade-off.

The converse is also true: all factors had a similar and small individual effect on the original Daw paradigm. To see this, compare the score of the original task with 0 factors to the scores of all tasks with 1 factor. The strength of the effect in the original task was only 1.3 standard deviations removed from the tasks with 1 factor, and even slightly better than the 1-factor task with the smallest effect. Most importantly, even if each individual factor did not substantially increase the total effect compared to the original paradigm, their joint inclusion increased the strength of the relationship between model-based control and reward rate by a factor of approximately 230.

At least in theory, we have developed a paradigm that embodies an accuracy-demand trade-off between model-based control and reward rate. Next, we attempt to validate this paradigm by having human participants perform either a novel version of the two-step task with the improved features described above, or the original version of the two-step task as described by Daw and colleagues [8]. We predicted that measures of model-based planning in the novel, but not in the original, paradigm would show a positive correlation with the reward rate.

In addition, the comparison between these two paradigms allows us to test whether human participants spontaneously modulate the balance between model-free and model-based control depending on whether a novel task favors model-based control. So far, we have discussed the accuracy-demand trade-off uniquely as it is instantiated in the two-step task. However, if the novel paradigm embodies an empirical accuracy-demand trade-off, then the results of this study allow us to test whether the brain also computes a cost-benefit trade-off between the two systems. We predicted that average model-based control would be elevated in the novel paradigm, since planning was incentivized in this task [56].

### Experimental methods

#### Participants

Four hundred and six participants (range: 18–70 years of age; mean: 33 years of age; 195 female) were recruited on Amazon Mechanical Turk to participate in the experiment. Participants gave informed consent, and the Harvard Committee on the Use of Human Subjects approved the study.

#### Materials and procedure

One hundred and ninety-nine participants completed 125 trials of the novel two-step reinforcement-learning task. The structure of the task was based on the procedure developed in the previous section. The remaining two hundred and seven participants completed 125 trials of the two-step with the original Daw structure [8]. We embedded both tasks in a cover story in order to make it more engaging for participants [31].

**Fig 15:**
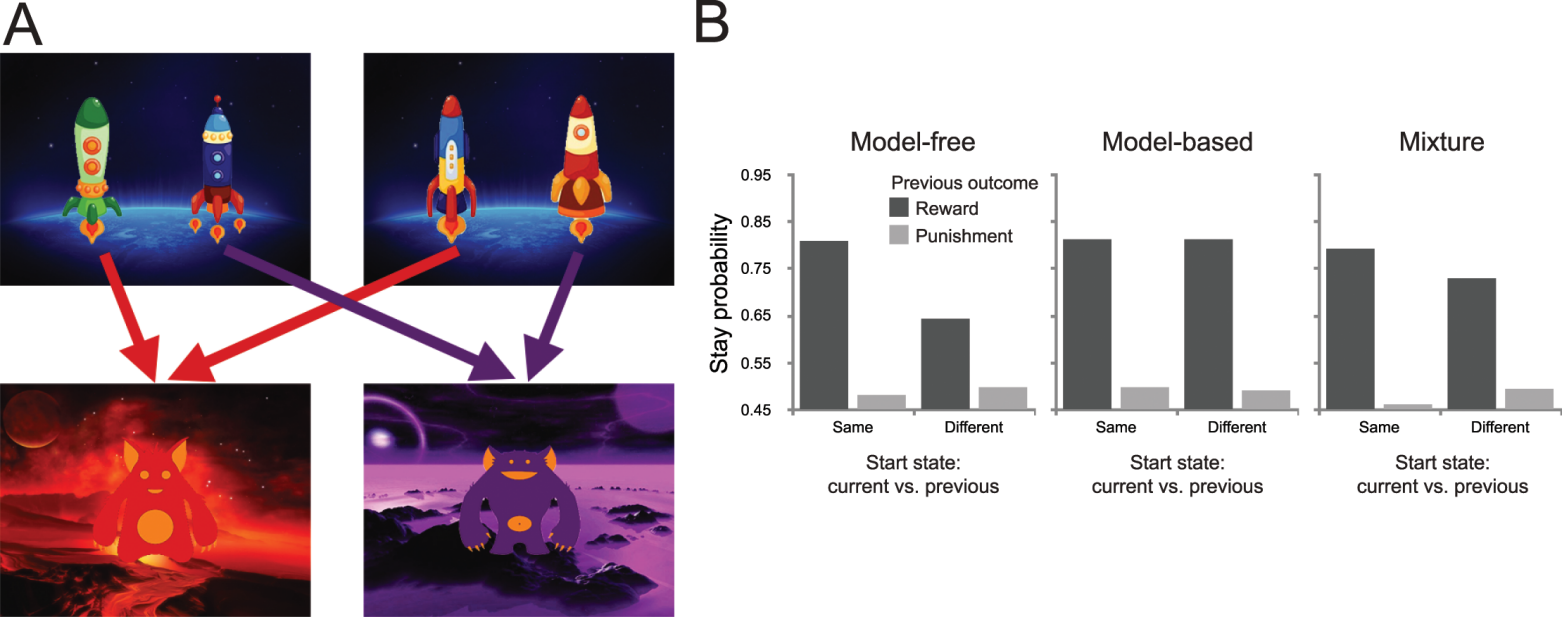
Design of the novel two-step task. (A) State transition structure of the paradigm. At the first stage, participants choose between one of two pairs of spaceships. Each choice deterministically leads to a second-stage state that was associated with a reward payoff that changed slowly according to a random Gaussian walk over the duration of the experiment. Note that the choices in the two different first-stage states are essentially equivalent. (B) Predicted behavior from the generative reinforcement-learning model of this task (using median parameter estimates, and *w* = 0.5 for the agent with a mixture of strategies). Note that in this task the model does not produce qualitatively different behavior for the different systems as reported in Fig 5. Instead, the differences in behavior are subtler, and therefore differences in strategy arbitration are better captured using model-fitting techniques.

*Novel paradigm*. Every trial in the novel two-step task consisted of two stages (Fig 15A). Each trial would start randomly in one of two possible first-stage states. In both, a pair of ‘spaceships’ appeared side by side on a blue earth-like planet background. Participants were told they had to choose between these two spaceships to fly to one of two different planets. The choice between the left- and right-hand spaceship had to be made using the “F” or “J” button keys within 2000ms. After a choice was made, the selected spaceship was highlighted for the remainder of the response period. The positions of the spaceships were randomly selected on each trial. Depending on the choice of spaceship, the participants would then deterministically transition to one of two second-stage states, a purple or a red planet. The spaceship selected in the first-stage was displayed at the top of the screen in this planet. On each planet, participants found an alien that ‘mines’ from a ‘space mine’. These mines act as the second-stage bandits. Participants were told that sometimes the aliens were in a good part of the mine and they paid off a certain number of points or ‘space treasure’, whereas at other times the aliens were mining in a bad spot, and this yielded negative points or ‘antimatter’. The payoffs of these mines slowly changed over the course of the experiment. Even though there was only one choice available at the second-stage planets, participants were instructed that they were to press the space bar within 2000ms in order to receive the reward. One of these reward distributions was initialized randomly within a range of -4 points to -1 points, and the other within a range of +1 to +5 points. Then, they varied according to a Gaussian random walk (*σ* = 2) with reflecting bounds at -4 and +5 for the remainder of the experiment. A new set of randomly drifting reward distributions was generated for each participant. At the end of the experiment, participants were given 1¢ for every two points they earned.

The most important feature of the task is that the spaceships at the first states were essentially equivalent. For each pair, one spaceship always led to the red planet and alien, whereas the other always led to the purple planet and alien. Because of this equivalence, we were able to dissociate model-based and model-free contributions to choice behavior, since only the model-based system generalizes across the equivalent start state options by computing each action’s value as its expected future reward. Therefore, model-based and model-free strategies make qualitatively different predictions about how second-stage rewards influence first-stage choices on subsequent trials. Specifically, for a pure model-based learner, each outcome at the second stage should affect first-stage preferences on the next trial, regardless of whether this trial starts with the same or the other pair of spaceships. In contrast, under a pure model-free strategy a reward obtained after one pair of spaceships should not affect choices between the other pair.

*Daw paradigm*. The Daw two-step task used the same buttons, timing, visual appearance, and counter balancing procedures as the novel paradigm, but the structure of the task matched that of the design in the original report (discussed in detail above). At the start of each trial, participants chose between a pair of spaceships. Depending on the choice of spaceship, the participants would then transition to one of two second-stage states, a purple or a red planet. Each spaceship traveled more frequently to one planet than to the other (70% versus 30%), and these transition probabilities were opposite for the two spaceships. On each planet, participants chose between pairs of aliens that mines from a space mine. Participants were told that sometimes the aliens were in a good part of the mine and they were more likely to deliver a piece of space treasure, whereas at other times the aliens were mining in a bad spot, and they were less likely to deliver space treasure. The payoffs of these mines slowly changed over the course of the experiment. One pair of aliens was initialized with probabilities of 0.25 and 0.75, and the other pair with probabilities of 0.4 and 0.6, after which they changed according to a Gaussian random walk (*σ* = 0.025) with reflecting bounds at 0.25 and 0.75 for the remainder of the experiment. A new set of randomly drifting reward distributions was generated for each participant. To equate average pay-off between conditions, participants were given 1¢ for every point they earned.

As explained in detail above, model-based and model-free strategies make qualitatively different predictions about how second-stage rewards influence first-stage choices on subsequent trials. Specifically, choice under a pure model-free strategy should not be affected by the type of transition (common vs. rare) observed on the previous trial (see Fig 2A), whereas pure model-based learners should base their choice on both the type of transition and whether a reward was observed on the previous trial (see Fig 2B).

Before completing the full task, participants were extensively trained on different aspects of the task. Participants who completed the novel paradigm first learned about the value of space treasure and antimatter, and the change in payoffs from both space mines by sampling rewards from two different aliens. Next, they learned about the deterministic transitions between spaceships and planets during a phase in which they were instructed to travel to one planet until accurate performance was reached. Participants who completed the Daw paradigm sampled from aliens with different reward probabilities, and were extensively instructed on the transition structure. Finally, both groups of participants practiced the full task for 25 trials. There was no response deadline for any of the sections of the training phase. The color of the planets and aliens in this phase were different from those in the experimental phase.

#### Reinforcement learning model and behavioral predictions

We used our reinforcement learning model of the novel task to produce behavioral predictions for a pure model-free and pure model-based decision maker, and an agent with a mixture between model-free and model-based control. This model was largely the same as before, with the exception of how the transition structures were learned.

Recall that participants that completed the novel paradigm performed a practice phase in which they were taught a set of deterministic transitions between the four spaceships and two different planets. Next, they were told that in the experimental phase, the rules and spaceships were the same as in the practice phase, but that there would be new planets. Therefore, we assumed that participants would assume equal probability of each spaceship traveling to one of the two planets, until they observed one transition for a first-stage state. After this observation, the model immediately infers the veridical transition structure for that first-stage state.

The participants that completed the Daw paradigm of the two-step task learned about the transition structure through instruction and direct experience in a practice phase with two different planets. They were also told that the rules and spaceships would be the same, but that the planets would be new. Therefore, we assumed that participants initially assumed equal probability of transitioning between the spaceships and the planets. Next, we characterized transition learning by assuming that participants chose between three possible transition structures as a function of how many transitions they observed between the states and actions: a flat structure with equal probabilities between all states and actions, or two symmetric transition structures with opposite transition probabilities of 70% and 30% between the two spaceships and planets.

As we have argued above, in our novel paradigm the differences in the probability of repeating the previous first-stage choice do not show a major qualitative difference between a purely model-based and model-free strategy, when plotted as a function of whether the previous start state is the same as or different from the current start state and whether a reward was obtained on the previous trial (Fig 15B). In fact, both a model-free and a mixture agent show an interaction between the two factors, start-state similarity and previous reward, with the likelihood of staying being higher if the current start state is similar the start state on the previous compared to when it was different, but still significantly bigger than chance. For the model-free agent, this reflects the presence of a highly rewarding action that the model-free learner learns to approach (for a detailed analysis, see [9]). This erosion of the qualitative predictions afforded by a stay/switch analysis is enhanced in these simulations compared to the original Doll investigation (Fig 5), presumably because reward observations in the current task carry more consequential information for behavior.

The lack of qualitative differences in single-trial staying behavior between the model-free and mixture strategies places special importance on model-fitting to quantify the balance between habit and control. Not only does model-fitting incorporate an influence of all previous trials on choice, but it also provides a numerical value for the relative weighting of model-based and model-free strategies (the *w* parameter).

In order to demonstrate that standard model-fitting procedures are sufficient to robustly estimate *w* on a per-participant basis, we generated data from 200 agents with randomly selected reinforcement learning parameters and then estimated these parameters using the model-fitting procedure described below. This method, described in more detail in the Supporting Information, yielded substantial correlations between the true and estimated parameters (including *w*, *r* = 0.68), validating our approach (S1 Text).

An alternative way to correct for the influence of reward in the previous trials is by predicting ‘staying’ behavior through a multilevel logistic regression analysis that accounts for this influence with a predictor that incorporates behavior about the outcome of the previous choice [9, 10]. The Supporting Information describes this method in detail; in brief, it produced qualitatively similar results to the model fitting procedure (S2 Text).

#### Model fitting

In order to estimate each participant’s weighting parameter, we fitted one of two reinforcement learning models to each participant’s data, dependent on which task they completed. This model was equivalent to the models described above, with the exception for the input into the softmax decision rule:

$$P(a_{i,t}=a|s_{i,t})=\frac{exp(\beta [Q_{net}(s_{i,t},a)+\pi ∙rep(a)+\rho ∙resp(a)])}{\sum_{a\prime }exp(\beta [Q_{net}(s_{i,t},a^{\prime })+\pi ∙rep(a^{\prime })+\rho ∙resp(a\prime )])}$$

where the indicator variable rep(*a*) is defined as 1 if *a* is a first-stage action and is the same one as was chosen on the previous trial, zero otherwise. Multiplied with the ‘stickiness’ parameter *π*, this captures the degree to which participants show perseveration (*π* > 0) or switching (*π* < 0) at the first stage. The indicator variable resp(*a*) is defined as 1 if *a* is a first-stage action selecting the same response key as the key that was pressed on the previous trial, zero otherwise. Multiplied with the ‘response stickiness’ parameter *ρ*, this captures the degree to which participants repeated (*ρ* > 0) or alternated (*ρ* < 0) key presses at the first stage. We introduced this parameter since the spaceship’s positions were not fixed, hence participants could show perseveration in spaceship choices, button presses, or both.

We used maximum *a posteriori* estimation with empirical priors, implemented using the *mfit* toolbox [54] parameters to fit the free parameters in the computational models to observed data for each participant separately. Based on prior work [54], we used weak priors for the distributions for the inverse temperature, *β ~* Gamma(4.82, 0.88), and stickiness parameters, *π, ρ ~ N*(0.15, 1.42), and flat priors for all other parameters. To avoid local optima in the estimation solution, we ran the optimization 25 times for each participant with randomly selected initializations for each parameter.

*Correlation analysis*. In order to assess the relationship between model-based control and reward in our novel paradigm, we computed the Pearson correlation coefficient between the estimated weighting parameter and reward rate obtained in the task. However, since we generated new sets of drifting rewards for each participant, baseline differences in average reward might weaken this correlation. Therefore, we calculated the difference between actual reward and average chance performance for each participant, and used this as the measure of reward obtained to correlate with the weighting parameter. For both tasks, chance performance was computed as the average value across the reward distributions.

#### Exclusion criteria

Participants were excluded from analysis if they timed out on more than 20% of all trials (more than 25), and we excluded all trials on which participants timed out (average 2.7%). After applying these criteria, data from 381 participants were submitted to the model-fitting procedure.

## Results

### Behavioral performance

**Fig 16:**
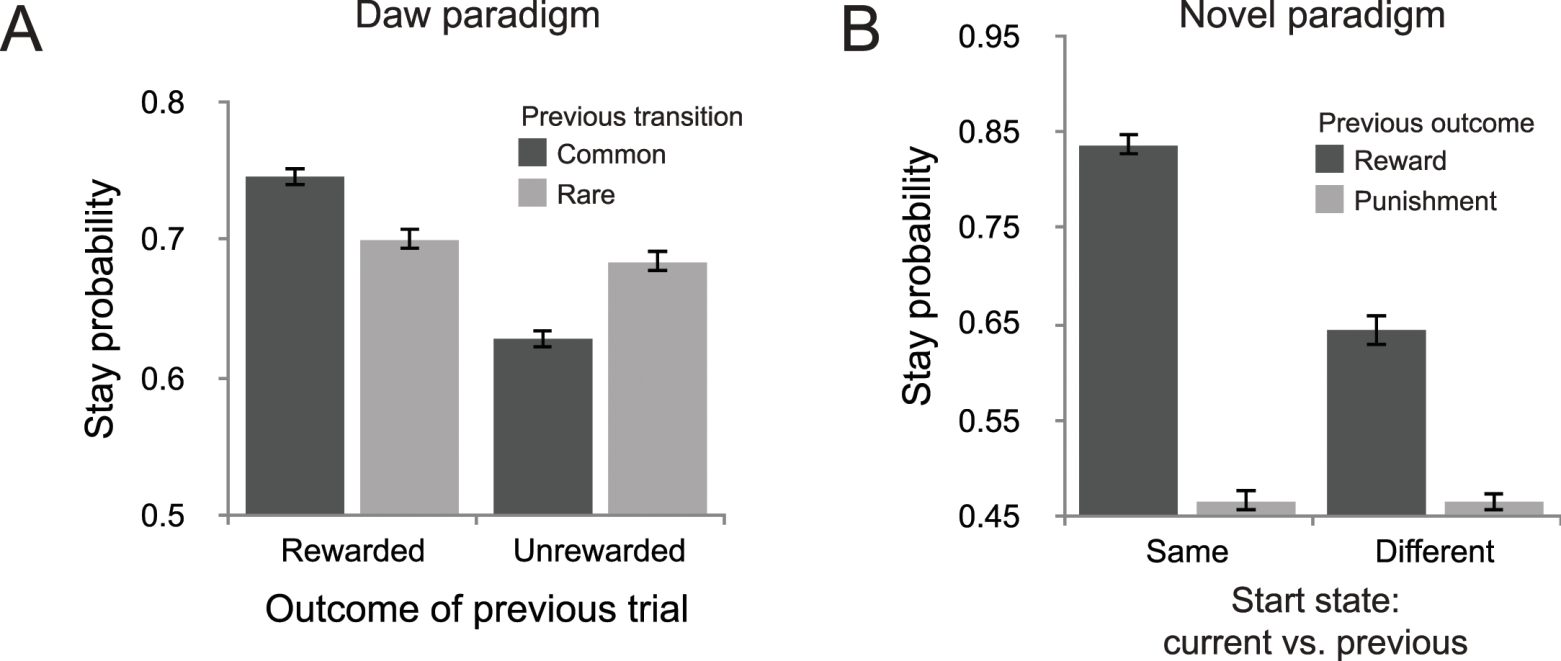
Behavioral performance on the two-step tasks. (A) Behavioral performance on the Daw task showed both a main effect of previous outcome and an interaction between previous outcome and transition type, suggesting that behavior showed both model-based and model-free strategies. (B) Behavioral performance on the novel paradigm showed a significant difference in stay behavior between same and different start states conditions after a reward, suggesting that behavior was not fully model-based. Error bars indicate within-subject SEM.

For the participants who completed the Daw task, we found that a reward on the previous trial increased the probability of staying with the previous trial’s choice [*t*(196) = 7.70, *p* < 0.001; Fig 16A], but that this effect interacted with the type of transition on the previous trial [*t*(196) = 5.38, *p* < 0.001]. This result replicates the basic finding on the original two-step confirming that participants used both model-based and model-free strategies.

For the participants who completed the new paradigm, we found that a positive reward on the previous trial significantly enhanced staying behavior from chance for both similar and different current start states, (*p* < 0.001 for both effects), but this effect was larger for the same compared to the different start state condition [*t*(183) = 9.64, *p* < 0.001; Fig 16B]. This pattern of behavior suggests that the participants did not employ a pure model-based strategy (compare with Fig 15B). However, as described above, it does not allow us to assess the relative contributions of model-based and model-free strategies to control based on these raw stay probabilities: both a purely model-free agent and an agent with a mixture of model-based and model-free strategies choices are predicted to show an increased stay probability after a win in a different start state, since a reward is indicative of history of recently reward trials.

### Model fits

The reinforcement learning models described above incorporates the (decayed) experience on all previous trials to choice and is better able to dissociate the contributions of the two strategies. This model consists of a model-free system that updates action values using temporal-difference learning and model-based system that learns the transition model of the task and uses this to compute action values online. The weighting parameter *w* determines the relative contribution between model-based and model-free control. The stickiness parameters *π* and *ρ* capture perseveration on either the response-level or the stimulus-choice.

We first investigated whether the inclusion of either stickiness parameter (*π* and *ρ*) was justified by comparing both the Bayesian Information Criterion (BIC) and Akaike Information Criterion (AIC), for models that included none, one, or both parameters for both tasks separately (see S1 Table). For the Daw task, we found that both goodness-of-fit measures favored a model that included both stickiness parameters. For the novel task, the BIC favored a model with response stickiness but not stimulus stickiness included, whereas the AIC favored a model that included both stickiness parameters. We decided to favor the more parsimonious model without stimulus stickiness, and parameter fits from this model will be reported in the following, but the results did not qualitatively change when the stimulus stickiness parameter was included.

Second, we used model comparison with both goodness-of-fit measures to analyze whether the hybrid model including the *w* parameter fit the data better than either a pure model-based or model-free model (see S2 Table). For the Daw task, we found that the AIC favored the hybrid model, but that the BIC favored the pure model-free model. However, there have been many reports in the literature that justify the inclusion of the weighting parameter for this task [8], and so we adopt the hybrid model for consistency with prior work. (Note also that it would be impossible to assess the relationship between model-based control and reward without using the hybrid model). For the new task, we found that both BIC and AIC favored the hybrid model compared to the pure model-based and model-free models. This suggests that human performance in the new paradigm is characterized by a mixture of model-based and model-free strategies.

In summary, the model fits presented below used all six free parameters for the participants that completed the Daw paradigm, but omitted the stimulus stickiness parameters for the participants that completed the novel paradigm. These parameter estimates and their quartiles are depicted in Table 1.

**Table 1:** Best-fitting parameter estimates shown as median plus quartiles across participants.

Paradigm	Predictor	*β*	*α*	*λ*	*π*	*ρ*	*w*
Daw	25^th^ percentile	2.35	0.11	0.25	0.03	-0.03	0.00
	Median	3.35	0.34	0.65	0.21	0.05	0.27
	75^th^ percentile	3.88	0.57	1.00	0.4	0.19	0.66
Novel	25^th^ percentile	0.51	0.01	0.07		-0.29	0.04
	Median	0.72	0.67	0.62		-0.06	0.48
	75^th^ percentile	3.31	1.00	1.00		0.14	0.85

Across participants, we found that the median weighting parameter *w* was 0.27 for the Daw paradigm and 0.48 for the novel paradigm, indicating that both strategies were mixed in the population for both tasks. However, we found that model-based control was significantly higher for participants in the novel paradigm compared to the Daw paradigm [Wilcoxon two-sample rank sum test, *z* = 3.31, *p* < 0.001], suggesting that the existence of the accuracy-demand trade-off in the novel paradigm induced a shift towards model-based control.

**Fig 17:**
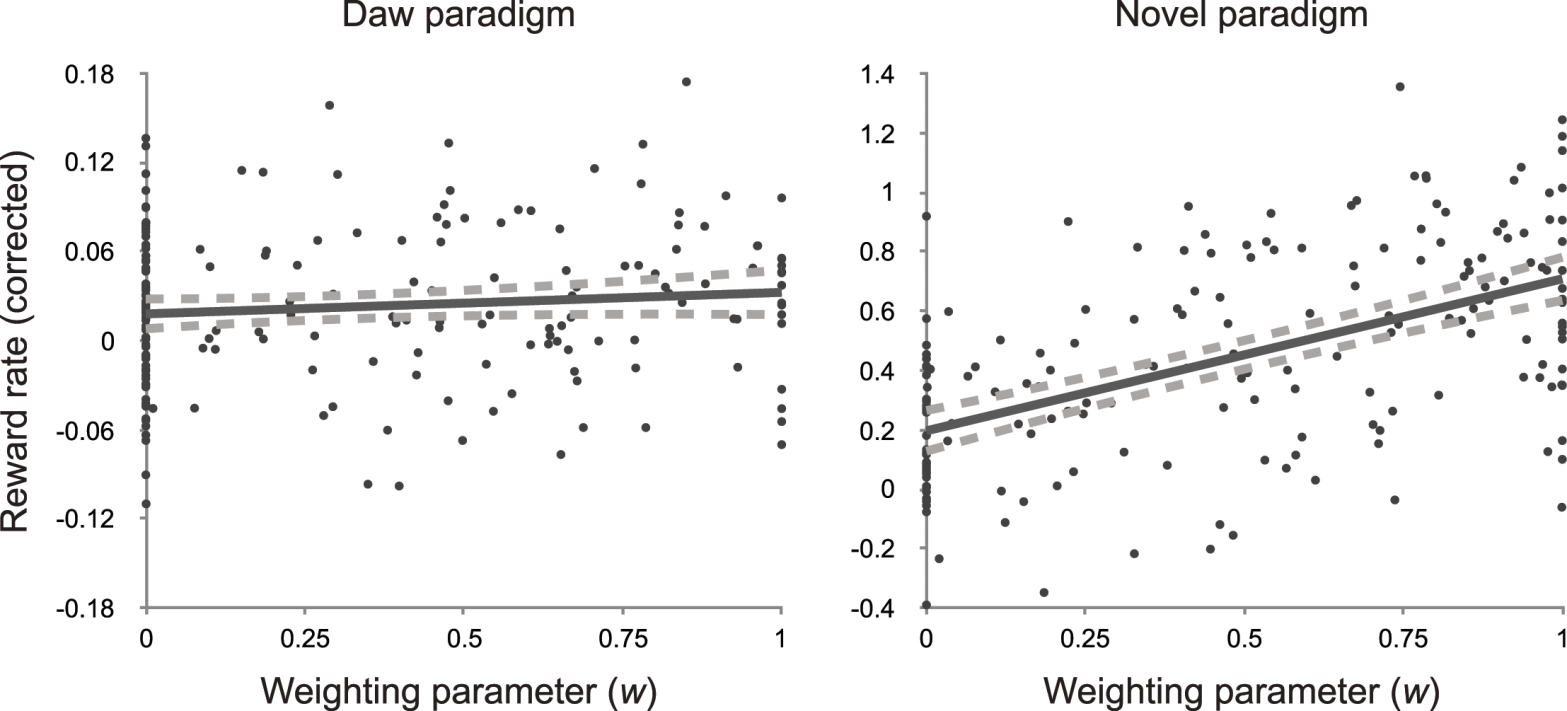
Relationship between the estimated weighting parameters and adjusted reward rate in the Daw and novel two-step paradigms. We found a positive correlation in the novel paradigm, but not in the original paradigm, suggesting that we successfully established a tradeoff between model-based control and reward in the two-step task. Dashed lines indicate the 95% confidence interval.

Of greatest relevance to our present aims, we found that the weighting parameter was positively related to our measure of the reward rate that controlled for average chance performance for the novel task (*r* = 0.55, *p* < 0.001), but not for the Daw paradigm (*r* = 0.10, *p* = 0.15; Fig 17). A subsequent multiple regression showed that this relationship was significantly different between groups [*t*(377) = 4.71, *p* < 0.001].

Next, in order to quantify the average gain in points across the entire range of *w* for both tasks, we ran a set of linear regression analyses predicting the reward rate from the weighting parameters for both tasks. For the Daw task, we found a predicted reward rate of 0.52 for *w* = 0 (i.e., the intercept), and an increase of 0.002 on top of this for *w* = 1 (i.e., the slope), indicating a 0.42% increase in points (to 0.525). For the novel task, we found a predicted reward rate of 0.58 for *w* = 0, and an increase of 0.67 on top of this for reward rate *w* = 1, indicating a 215% increase in points (to 1.25). When we computed these slopes using the corrected reward rates, subtracting the average value of each participant’s reward distribution from their reward rate, we found an average increase in reward rate of 0.01 across the range of the weighting parameter for the Daw task, and an average increase in reward rate of 0.51 for the novel task.

These results validate the accuracy-demand trade-off of the novel two-step paradigm, and also demonstrate that the original Daw two-step paradigm does not embody such a trade-off.

## Discussion

The distinction between planning and habit lies at the core of behavioral and neuroscientific research, and plays a central role in contemporary dual process models of cognition and decision making. Modern reinforcement learning theories formalize the distinction in terms of model-based and model-free control, bringing new computational precision to the long-recognized trade-off between accuracy and demand in decision making. In principle, the model-based strategy attains more accurate performance through increased effort relative to the computationally inexpensive but more inaccurate model-free strategy.

Yet, building on prior work [9], we provide an exhaustive demonstration that the hallmark task for dissociating model-based and model-free control—the Daw two-step paradigm (Fig 3A) and several related variants of this task (Figs 4 and 5)—do not embody a trade-off between accuracy and demand across a wide range of parameter space. Using simulations of reinforcement learning agents on variants of the two-step task, we have identified five features that reduce the reward associated with model-based control to such a degree that pure model-based and model-free agents obtain equivalent reward (Fig 13). By systematically eliminating these features from the task, we developed a novel variant that shows a strong relationship between model-based control and reward both in simulations and in experimental data. In addition to providing new insight into the affordances of distinct experimental paradigms, our findings demonstrate that the magnitude of the accuracy-demand trade-off varies greatly with the specific features of any given task.

First, we found that the trade-off depends on highly distinguishable reward probabilities. Broadening the range of possible reward probabilities (from 0 to 1) contributed a small, but measurable effect on the relationship between model-based control and reward (Simulation 1, Fig 6B). Second, we found that the trade-off depends on the rate of change of the second-stage reward probabilities. Our analyses indicates that the rate of change in the original paradigm was too slow to elicit a reliable accuracy-demand trade-off, because it allowed the model-free strategy to integrate sufficient information over trials to match the performance of the model-based strategy (Fig 7A). Based on this analysis, we showed that a task with larger drift rate produced a stronger relationship between model-based control and reward (Simulation 2; Fig 7B). Third, the trade-off can be limited by the presence of stochastic transitions. In the original two-step task, model-based choices do not always lead to the desired second-stage state, since this paradigm includes rare transitions from the first to the second stage, reducing the efficacy of model-based control. A new transition structure, using deterministic transitions from two different starting states, avoids this issue, and substantially strengthens the accuracy-demand trade-off (Simulation 3; Fig 8A). Fourth, the trade-off is limited when the environment contains a large number of actions bounded by the same rewarded probabilities. Specifically, by reducing the number of second-stage choice options, the average difference in value between the optimal choices of the two second-stage states is increased, which allows the model-based advantage at the first stage to emerge more distinctly. This change to the paradigm further strengthens the accuracy-demand trade-off (Simulation 4; Fig 9). Fifth, the trade-off is limited under conditions of high uncertainty about the reward value of actions. Specifically, we found that the stochastic reward observations in this task do not carry enough information about the value of the associated stimuli. Subsequently, removing the binomial noise from the reward distributions leads to a substantial increase in the strength of the accuracy-demand trade-off in this paradigm (Simulation 5; Fig 11). Moreover, we find that these factors have a superadditive effect on the relationship between model-based control and reward: All five changes to the task are required to establish a reliable accuracy-demand trade-off. We experimentally confirmed these theoretical predictions, demonstrating that the empirical estimate of model-based control in the new task was correlated with reward rate across participants.

It is likely that more than these five factors alone moderate the effect of model-based control on accuracy. For example, in the Akam version of the two-step task, rewards alternate between blocks of opposite reward probabilities, so that one option strictly dominates the other until the next alternation is implemented. As discussed, this change to the paradigm resulted in a strong trade-off between control and reward in a selective region of parameter space. It is plausible that there are alternative versions of the two-step task that embody an even stronger trade-off than those discussed here, and we look forward to a comparison of how those relate to the current paradigm.

In addition to the difference in the strength of the accuracy-demand trade-off between paradigms, we also found that novel two-step task elicited greater average model-based control in our participants than the original Daw two-step task. This result is one of the first pieces of behavioral evidence suggesting an adaptive trade-off between model-based and model-free control. Put simply, participants reliably shifted towards model-based control when this was a more rewarding strategy. This may indicate that participants store “controller values” summarizing the rewards associated with model-based and model-free control. However, there are alternative explanations for this result. For example, it is possible the presence of deterministic transition structure or the introduction of negative reward induced increased model-based control triggered by a Pavlovian response to these types of task features. In other words, the increase in planning might not a reflect motivational trade-off, but rather a simple decision heuristic that does not integrate computational demand and accuracy. Future investigations, where task features and reward are independently manipulated, will be able to provide more conclusive evidence that people adaptively weigh the costs and benefits of the two strategies against each other.

Although the original Daw two-step task does not embody an accuracy-demand trade-off, choice behavior on this task nonetheless reflects a mixture of model-based and model-free strategies. Furthermore, the degree of model-free control on this task is predicted by individual difference measures such as working memory capacity [23], cognitive control ability [24], processing speed [29], age [20, 31], extraversion [30], and even psychiatric pathology [11, 33, 34]. This discrepancy demands explanation. Why does the original task, without a motivational trade-off, still yield meaningful and interpretable results? One possibility is that, in the absence of a reliable signal from the environment, behavior on this task reflects participants’ belief about how model-based control relates to reward maximization in the real world (where the trade-off is presumably more pervasive). Another possibility is that the extensive training of participants on the transition structure of the experiment induces them to assume they should be using it during task performance. In this sense, the absence of a trade-off is not problematic for mapping out individual differences that co-vary with the use of model-based control.

This analysis can help explain the types of experimentally induced shifts in control allocation that have been reported using the two-step task, as well as those that have not. Prior research has demonstrated several factors that increase the control of model-free strategies on decision making. Control shifts to the model-free system with extensive experience [57], under cognitive load [22], and after the induction of stress [23, 28]. Such shifts are rational insofar as there is no advantage to model-based control in the task. Notably, however, few studies report factors that *increase* the use of model-based control. The exception to this rule is a study in which the underlying neural mechanism was altered by administering dopamine agonists after which control shifted to the model-based system [12]. Apart from this report, no other studies have successfully increased model-based control in the two-step task. Our simulation results suggest an explanation: in the original version of the two-step task, planning behavior does not improve reward, and so there is no incentive to increase the contribution of the model-based system.

Our novel paradigm opens up the possibility of studying the neural mechanism underlying the trade-off between model-based and model-free control. The first and most influential neuroimaging study of the two-step task [8] focused on the neural correlates of “reward prediction error” (the difference between expected and observed reward) that is used by both the model-based and model-free controllers. A host of previous research shows that model-free reward prediction errors are encoded in the striatum [36]. The results of Daw and colleagues [8] were in line with this finding; the reward prediction errors of the model-free system correlated with signal in the striatum. However, despite the distinct computational features of the two systems, the model-based reward prediction errors recruited a similar, indistinguishable, region of the striatum (see also [13]). Our recent simulations may shed light on this surprising finding, insofar as model-based system was not appropriately incentivized. An important area for future research is to identify the neural correlates of model-based control under conditions where it obtains a higher average rate of reward than does model-free control.

One potential limitation of the current paradigm is that it does not afford a simple qualitative characterization of model-based versus model-free control based exclusively on the relationship between reward (vs. punishment) on one trial and a consistent (vs. inconsistent) behavioral policy on the subsequent trial. As depicted in Fig 15B, both strategies predict an increased likelihood of behavioral consistency after a reward in either start state, but also a higher probability of consistency when the current start state is the same as in the previous trial compared to when the current start state is different. Our results reinforce this point. Even though the raw consistency behavior was not able to distinguish between the pure model-free and mixture strategies, our model-fitting procedure showed that most participants employed both model-based and model-free strategies.

Indeed, our exploration of this point revealed an apparent mystery and suggests a potentially illuminating explanation. Although our full model fits of participant data indicate a high degree of model-based control, this trend is not at all evident in their raw stay probabilities, conditioned on reward in the previous trial. Not only do we fail to find the high staying probability we would expect for trials on which the associated stage-one choice was previously rewarded (assuming some influence of model-based control), in fact we find an even lower stay probability than would be expected given a computational model of pure model-free control. How can we explain this divergence between our empirical result and the predictions of our generative model? Recent work on the influence of working memory capacity on reinforcement learning may shed some light on this puzzling finding. Collins and Frank [58] show that the performance accuracy on a reinforcement learning task varied as a function of the number of stimuli that had to be remembered (the load) and the delay between repetitions of the same choice. Behavior in the current task is likely to be subject to similar constraints, since the number of choice options (six) is well above the capacity limit reported by Collins and Frank [58]. Therefore, the smaller-than-predicted probability of staying after a reward in the different start state might be predicted be memory decay, since the average delay of seeing the stimuli in this state is strictly higher than in trials with the same starting state. Exploring these possibilities further, while beyond the scope of the present study, is a key area for further investigation.

Finally, we observed a shift in arbitration between model-based and model-free control when comparing the original and novel versions of the two-step paradigm. Specifically, participants in the novel paradigm were more likely to adopt the model-based strategy compared to those who completed the Daw version of the task. This result is one of the first pieces of evidence that the people negotiate an accuracy-demand trade-off between model-based and model-free strategies, and is consistent with a large body of literature that suggests that increased incentives prime more intense controlled processing [56]. Though tantalizing, this result raises several new questions. For example, how does the brain adapt its allocation between model-based and model-free control? At what time scale is this possible? What is the appropriate computational account of arbitration between the two systems? What neural regions are involved in determining whether one should exert more model-based control? Future investigations, using a combination of neural, behavioral, and computational methods will aim at answering these questions.

### Conclusion

In recent years, the Daw two-step task has become the gold standard for describing the trade-off between accuracy (model-based control) and computational demand (model-free control) in sequential decision making. Our computational simulations of this task reveal that it does not embody such a trade-off. We have developed a novel version of this task that theoretically and empirically obtains a relationship between model-based control and reward (a proxy for the accuracy-demand trade-off). The current investigation reveals a critical role for computational simulation of predicted effects, even if these appear to be intuitive and straightforward. It also introduces a new experimental tool for behavioral and neural investigations of cost-benefit trade-offs in reinforcement learning. Finally, it opens new avenues for investigating the features of specific tasks, or domains of task, that favor model-based over model-free control.

## Supporting Information

S1 Fig The influence of the drift rate in the two-step task across a broad range of RL parameters. We found that the size of the drift rate affected the strength between model-based control and reward in a non-monotonic fashion, with the largest effect found at moderate values of the drift rate (0.1–0.3) and with a broad reward probability range. Importantly, the results of this analysis shows that this effect was not only found in the particular parameterization depicted in Fig 8 in the main text, but also across a broad range of learning rates (α) and inverse temperatures (β).
(EPS)

S2 Fig Volume under the surface for all 32 tasks generated by the 5 binary factors discussed in this paper for agents with eligibility decay parameter λ = 0 and λ = 1. Each dot represents the volume under the surface of linear regression coefficients for one task, and is plotted as a function of the number of ‘beneficial’ factors that are included in each task’s design. The gray line represents the average increase in the strength of the relationship between model-based control and reward. These results are qualitatively identical to those reported in Fig 13, suggesting that λ does not reliably affect the strength of the accuracy-efficiency tradeoff.
(EPS)

S1 Text Reliability analysis for the model-fitting procedure. (DOCX)

S2 Text Multi-level logistic regression analyses. (DOCX)

S1 Table Model comparison for the full hybrid model and the hybrid model without choice perseveration parameters. (DOCX)

S2 Table Model comparison for the hybrid model and pure model-based and model-free models. (DOCX)

## References

[1] Dickinson A. Actions and habits: The development of behavioural autonomy. Philosophical Transactions of the Royal Society B: Biological Sciences. 1985; 308: 67–78.

[2] Sloman SA. The empirical case for two systems of reasoning. Psychological Bulletin. 1996; 119: 3–22.

[3] Kahneman D. A perspective on judgment and choice: Mapping bounded rationality. American Psychologist. 2003; 58: 697–720. 14584987 10.1037/0003-066X.58.9.697

[4] Fudenberg D, Levine DK. A dual self model of impulse control. American Economic Review. 2006; 96: 1449–76. 29135208 10.1257/aer.96.5.1449

[5] Balleine BW, O'Doherty J. Human and rodent homologies in action control: Corticostrialtal determinants of goal-directed and habitual action. Neuropsychopharmacology. 2009; 35: 48–69. 10.1038/npp.2009.131 PMC3055420 19776734

[6] Dolan RJ, Dayan P. Goals and habits in the brain. Neuron. 2013; 80: 312–25. 10.1016/j.neuron.2013.09.007 24139036 PMC3807793

[7] Daw ND, Niv Y, Dayan P. Uncertainty-based competition between prefrontal and dorsolateral striatal systems for behavioral control. Nature Neuroscience. 2005; 8: 1704–11. 16286932 10.1038/nn1560

[8] Daw ND, Gershman SJ, Seymour B, Dayan P, Dolan RJ. Model-based influences on humans' choices and striatal prediction errors. Neuron. 2011; 69: 1204–15. 10.1016/j.neuron.2011.02.027 21435563 PMC3077926

[9] Akam T, Costa R, Dayan P. Simple Plans or Sophisticated Habits? State, Transition and Learning Interactions in the Two-Step Task. PLoS computational biology. 2015; 11: e1004648–25. 10.1371/journal.pcbi.1004648 26657806 PMC4686094

[10] Smittenaar P, FitzGerald THB, Romei V, Wright ND, Dolan RJ. Disruption of dorsolateral prefrontal cortex decreases model-based in favor of model-free control in humans. Neuron. 2013; 80: 914–9. 10.1016/j.neuron.2013.08.009 24206669 PMC3893454

[11] Worbe Y, Palminteri S, Savulich G, Daw ND, Fernandez-Egea E, Robbins TW, et al Valence-dependent influence of serotonin depletion on model-based choice strategy. Molecular Psychiatry. 2015: 1–6. 25869808 10.1038/mp.2015.46 PMC4519524

[12] Wunderlich K, Smittenaar P, Dolan R. Dopamine enhances model-based over model-free choice behavior. Neuron. 2012; 75: 418–24. 10.1016/j.neuron.2012.03.042 22884326 PMC3417237

[13] Deserno L, Huys QJM, Boehme R, Buchert R, Heinze H-J, Grace AA, et al Ventral striatal dopamine reflects behavioral and neural signatures of model-based control during sequential decision making. Proceedings of the National Academy of Sciences. 2015; 112: 1595–600. 10.1073/pnas.1417219112 PMC4321318 25605941

[14] Doll BB, Bath KG, Daw ND, Frank MJ. Variability in dopamine genes dissociates model-based and model-free reinforcement kearning. Journal of Neuroscience. 2016; 36: 1211–22. 10.1523/JNEUROSCI.1901-15.2016 26818509 PMC4728725

[15] Doll BB, Duncan KD, Simon DA, Shohamy D, Daw ND. Model-based choices involve prospective neural activity. Nature Neuroscience. 2015; 18: 767–72. 10.1038/nn.3981 25799041 PMC4414826

[16] Doll BB, Hutchison KE, Frank MJ. Dopaminergic genes predict individual differences in susceptibility to confirmation bias. Journal of Neuroscience. 2011; 31: 6188–98. 10.1523/JNEUROSCI.6486-10.2011 21508242 PMC3098533

[17] Morris LS, Kundu P, Dowell N, Mechelmans DJ, Favre P, Irvine MA, et al Fronto-striatal organization: Defining functional and microstructural substrates of behavioural flexibility. CORTEX. 2016; 74: 118–33. 10.1016/j.cortex.2015.11.004 26673945 PMC4729321

[18] Smittenaar P, Prichard G, FitzGerald THB, Diedrichsen J, Dolan RJ. Transcranial direct current stimulation of right dorsolateral prefrontal cortex does not affect model-based or model-free reinforcement learning in humans. PLoS ONE. 2014; 9: e86850–8. 10.1371/journal.pone.0086850 24475185 PMC3901733

[19] Economides M, Kurth-Nelson Z, Lübbert A, Guitart-Masip M, Dolan RJ. Model-based reasoning in humans becomes automatic with training. PLOS Computational Biology. 2015; 11: e1004463–19. 10.1371/journal.pcbi.1004463 26379239 PMC4588166

[20] Eppinger B, Walter M, Heekeren HR, Shu-Chen L. Of goals and habits: age-related and individual differences in goal-directed decision-making. Frontiers in Neuroscience. 2013; 7: 253 10.3389/fnins.2013.00253 24399925 PMC3871973

[21] Gillan CM, Otto AR, Phelps EA, Daw ND. Model-based learning protects against forming habits. Cognitive, Affective, & Behavioral Neuroscience. 2015; 15: 523–36. 10.3758/s13415-015-0347-6 PMC4526597 25801925

[22] Otto AR, Gershman SJ, Markman AB, Daw ND. The curse of planning: Dissecting multiple reinforcement-learning systems by taxing the central executive. Psychological Science. 2013; 24: 751–61. 10.1177/0956797612463080 23558545 PMC3843765

[23] Otto AR, Raio CM, Chiang A, Phelps E, Daw N. Working-memory capacity protects model-based learning from stress. Proceedings of the National Academy of Sciences USA. 2013; 110: 20941–6. 10.1073/pnas.1312011110 PMC3876216 24324166

[24] Otto AR, Skatova A, Madlon-Kay S, Daw ND. Cognitive control predicts use of model-based reinforcement learning. Journal of Cognitive Neuroscience. 2015; 27: 319–33. 10.1162/jocn_a_00709 25170791 PMC4387848

[25] Dezfouli A, Balleine BW. Actions, action sequences and habits: Evidence that goal-directed and habitual action control are hierarchically organized. PLOS Computational Biology. 2013; 9: e1003364–14. 10.1371/journal.pcbi.1003364 24339762 PMC3854489

[26] Dezfouli A, Lingawi NW, Balleine BW. Habits as action sequences: hierarchical action control and changes in outcome value. Philosophical Transactions of the Royal Society of London Series B, Biological sciences. 2014; 369: 20130482–. 10.1098/rstb.2013.0482 25267824 PMC4186235

[27] Friedel E, Koch SP, Wendt J, Heinz A, Deserno L, Schlagenhauf F. Devaluation and sequential decisions: linking goal-directed and model-based behavior. Frontiers in Human Neuroscience. 2014; 8: 587 10.3389/fnhum.2014.00587 25136310 PMC4120761

[28] Radenbach C, Reiter AMF, Engert V, Sjoerds Z, Villringer A, Heinze H-J, et al The interaction of acute and chronic stress impairs model-based behavioral control. Psychoneuroendocrinology. 2015; 53: 268–80. 10.1016/j.psyneuen.2014.12.017 25662093

[29] Schad DJ. Processing speed enhances model-based over model-free reinforcement learning in the presence of high working memory functioning. Frontiers in Psychology. 2014; 5: 1450 10.3389/fpsyg.2014.01450 25566131 PMC4269125

[30] Skatova A, Chan PA, Daw ND. Extraversion differentiates between model-based and model-free strategies in a reinforcement learning task. Frontiers in Human Neuroscience. 2015; 7: 525. 10.3389/fnhum.2013.00525 PMC3760140 24027514

[31] Decker JH, Otto AR, Daw ND, Hartley CA. From creatures of habit to goal-directed learners: Tracking the developmental emergence of model-based reinforcement learning. Psychological Science. in press. 10.1177/0956797616639301 PMC4899156 27084852

[32] Sharp ME, Foerde K, Daw ND, Shohamy D. Dopamine selectively remediates &model-based& reward learning: a computational approach. Brain. 2015; 139: 355–64. 10.1093/brain/awv347 26685155 PMC5868097

[33] Voon V, Baek K, Enander J, Worbe Y, Morris LS, Harrison NA, et al Motivation and value influences in the relative balance of goal-directed and habitual behaviours in obsessive-compulsive disorder. Translational Psychiatry. 2015; 5: e670–8. 10.1038/tp.2015.165 26529423 PMC5068758

[34] Voon V, Derbyshire K, ck CRu, Irvine MA, Worbe Y, Enander J, et al Disorders of compulsivity: a common bias towards learning habits. Molecular Psychiatry. 2014; 20: 345–52. 10.1038/mp.2014.44 24840709 PMC4351889

[35] Gillan CM, Kosinski M, Whelan R, Phelps EA, Daw ND. Characterizing a psychiatric symptom dimension related to deficits in goal-directed control. eLife. in press. 10.7554/eLife.11305 PMC4786435 26928075

[36] Schultz W, Dayan P, Montague PR. A neural substrate of prediction and reward. Science. 1997; 275: 1593–9. 9054347 10.1126/science.275.5306.1593

[37] Sutton RS, Barto AG. Reinforcement Learning: An Introduction. Cambridge, MA: MIT Press; 1998 12 22.

[38] Pezzulo G, Rigoli F, Chersi F. The Mixed Instrumental Controller: Using Value of Information to combine habitual choice and mental simulation. Frontiers in Psychology. 2013; 4: 92 10.3389/fpsyg.2013.00092 23459512 PMC3586710

[39] Deserno L, Wilbertz T, Reiter A, Horstmann A, Neumann J, Villringer A, et al Lateral prefrontal model-based signatures are reduced in healthy individuals with high trait impulsivity. Translational Psychiatry. 2015; 5: e659 10.1038/tp.2015.139 26460483 PMC4930122

[40] Gläscher J, Daw N, Dayan P, O'Doherty J. States versus rewards: dissociable neural prediction error signals underlying model-based and model-free reinforcement learning. Neuron. 2010; 66: 585–95. 10.1016/j.neuron.2010.04.016 20510862 PMC2895323

[41] Gershman SJ, Markman AB, Otto AR. Retrospective revaluation in sequential decision making: A tale of two systems. Journal of Experimental Psychology: General. 2014; 143: 182–94. 23230992 10.1037/a0030844

[42] Cushman F, Morris A. Habitual control of goal selection in humans. Proceedings of the National Academy of Science. 2015. 10.1073/pnas.1506367112 PMC4653221 26460050

[43] Kool W, McGuire JT, Rosen ZB, Botvinick MM. Decision making and the avoidance of cognitive demand. Journal of Experimental Psychology: General. 2010; 139: 665–82. 20853993 10.1037/a0020198 PMC2970648

[44] Kurzban R, Duckworth AL, Kable JW, Myers J. An opportunity cost model of subjective effort and task performance. Behavioral and Brain Sciences. 2013; 36: 661–726. 10.1017/S0140525X12003196 24304775 PMC3856320

[45] Westbrook A, Kester D, Braver TS. What is the subjective cost of cognitive effort? Load, trait, and aging effects revealed by economic preference. PLOS ONE. 2013; 22: e68210. 10.1371/journal.pone.0068210 PMC3718823 23894295

[46] Keramati M, Dezfouli A, Piray P. Speed/accuracy trade-off between the habitual and the goal-directed processes. PLOS Computational Biology. 2011; 7: e1002055–21. 10.1371/journal.pcbi.1002055 21637741 PMC3102758

[47] Gershman SJ, Horvitz EJ, Tenenbaum JB. Computational rationality: A converging paradigm for intelligence in brains, minds, and machines. Science. 2015; 349: 273–8. 10.1126/science.aac6076 26185246

[48] Griffiths TL, Lieder F, Goodman ND. Rational use of cognitive resources: Levels of analysis between the computational and the algorithmic. Topics in Cognitive Science. 2015; 7: 217–29. 10.1111/tops.12142 25898807

[49] Payne JW, Bettman JR, Johnson EJ. Adaptive strategy selection in decision making. Journal of Experimental Psychology: Learning, Memory, and Cognition. 1988; 14: 534–52.

[50] Rieskamp J, Otto PE. SSL: A theory of how people learn to select strategies. Journal of Experimental Psychology: General. 2006; 135: 207–36. 16719651 10.1037/0096-3445.135.2.207

[51] Lee SW, Shimojo S, O'Doherty JP. Neural computations underlying arbitration between model-based and model-free Learning. Neuron. 2014; 81: 687–99. 10.1016/j.neuron.2013.11.028 24507199 PMC3968946

[52] Rummery G, Niranjan M. On-line Q-learning using connectionist systems. Cambridge University 1994.

[53] Simon DA, Daw ND. Environmental statistics and the trade-off between model-based and TD learning in humans. In: Shawe-Taylor J, Zemel R, Bartlett P, Pereira F, Weinberger K, editors. Advances in Neural Information Processing Systems. 242011. p. 127–35.

[54] Gershman SJ. Empirical priors for reinforcement learning models. Journal of Mathematical Psychology. 2016; 71: 1–6.

[55] Behrens TEJ, Woolrich MW, Walton ME, Rushworth MFS. Learning the value of information in an uncertain world. Nature Neuroscience. 2007; 10: 1214–21. 17676057 10.1038/nn1954

[56] Botvinick MM, Braver T. Motivation and cognitive control: From behavior to neural mechanism. Annual Review of Psychology. 2015; 66: 83–113. 10.1146/annurev-psych-010814-015044 25251491

[57] Daw ND, Shohamy D. The cognitive neuroscience of motivation and learning. Social Cognition. 2008; 26: 593–620.

[58] Collins AGE, Frank MJ. How much of reinforcement learning is working memory, not reinforcement learning? A behavioral, computational, and neurogenetic analysis. European Journal of Neuroscience. 2012; 35: 1024–35. 10.1111/j.1460-9568.2011.07980.x 22487033 PMC3390186

