# Supplementary material for: When Does Model-Based Control Pay Off?
Source: PLoS Comput Biol. 2016 Aug 26;12(8):e1005090. doi: 10.1371/journal.pcbi.1005090 (PMC5001643; doi:10.1371/journal.pcbi.1005090)
Supplement: S1 Text — (DOCX) [file pcbi.1005090.s003.docx]

**S1 Text. Reliability analysis for the model-fitting procedure**

Here, we report an analysis to test whether our model-fitting procedure could reliably dissociate model-based from model-free control (even though there was a lack of qualitative difference in single-trial staying behavior, see Figure 13B in the main text). In order to do so, we used the generative RL model of our novel two-step paradigm to simulate behavioral performance for 200 agents with randomly selected parameters. For each agent, we randomly sampled parameters from uniform distributions: $\left\{ \alpha, \lambda,w \right\}\sim U(0,+1)$, $\beta\sim U\left( 0,+2 \right)$.

Next, we used our model-fitting procedure to estimate parameters for each of our agent using MATLAB’s *patternsearch* function. To avoid local optima in the estimation solution, we ran 25 iterations for each agent with randomly selected starting positions for each parameter. We extracted from the fit with maximal log-likelihood. The final estimations for all parameters were extracted from the iteration with the maximal log-likelihood.

We found substantial correlations between the true and estimated values for submitted parameters. Most importantly, the weighting parameter *w* showed a strong and positive correlation, *r* = 0.68, *p* < 0.001, showing that our method is able to extract meaningful parameter estimates even when behavioral data do not qualitatively discriminate between different modes of processing. Correlations for the other parameters were as follows. Inverse temperature parameter *β*, *r* = 0.25, *p* < 0.001, learning rate parameter *α*, *r* = 0.82, *p* < 0.001, and eligibility trace decay parameter *λ*, *r* = 0.27, *p* < 0.001.
