# Supplementary material for: When Does Model-Based Control Pay Off?
Source: PLoS Comput Biol. 2016 Aug 26;12(8):e1005090. doi: 10.1371/journal.pcbi.1005090 (PMC5001643; doi:10.1371/journal.pcbi.1005090)
Supplement: S2 Text — (DOCX) [file pcbi.1005090.s004.docx]

**S2 Text. Multilevel logistic regression analyses**

In addition to the model fitting analyses reported in the main text, we also fit multilevel logistic regression models to the data from both participants that completed the Daw paradigm and those that completed the novel paradigm. These analyses were carried out using the lme4 package (http://cran.r-project. org/web/packages/lme4/index.html) in the R statistical language (http://www. r-project.org/). In order to measure individual difference in choice behavior, we modeled all coefficients as random effects, varying between participants around a group mean.

Analysis

*Novel paradigm*

For participants in the novel paradigm, we predicted whether the participants repeated the previous trial’s second-stage state (i.e., “staying behavior”) as a function of the rewards and similarity of the previous trial’s first-stage state. Specifically, the dependent variable was whether the current second-stage choice was the same as that on the previous trial. For each trial *i*, the predictors for this analysis were the amount of points on the previous trial (*r*_i-1_) and whether the previous starting state was the same or different from the current starting state (*same*_i_ = 1 or *same*_i_ = 0, respectively). In addition, in order to account for the influence of the prior reward history, we included a predictor that coded for the difference in possible reward between chosen and unchosen terminal states on the previous trial (*difference*_i-1_). The final multilevel regression model included these three predictors, their interactions and the intercept. We ran this analysis for the experimental data (*n* = 185). In addition, in order to gain more insight into the range of possibilities for this analysis, we ran the same analysis for two simulated sets of data that were generated using the dual-systems RL model of the novel paradigm. For each set, we matched one agent to each of our participants, copying their parameter fits except for the weighting parameter, which was set to *w* = 0 for one set (the model-free set), and *w* = 1 for the other (the model-based set).

In these regression analyses, the main effect of the previous reward (*r*_i-1_) represents the model-based contribution to choice, since it carries over to the next trial even when the start states are different (*same*_i_ = 0), whereas the interaction term *r*_i-1_ * *same*_i_ captures reward effects that are specific to the state in which they were received and therefore represent the model-free contribution to choice. We computed the difference between the model-based and model-free coefficients as an analogous term to the weighting parameter *w* in the computation model.

*Daw paradigm*

For the Daw task, we predicted whether the current trial’s first stage choice was the same the previous trial’s first-stage choice state (i.e., “staying behavior”) as a function of whether the previous trial produced a reward (*r*_i-1_) and what type of transition occurred on that trial (*common*_i_). The final multilevel regression model included these two predictors, their interactions and the intercept. We ran this analysis for the experimental data (*n* = 198). We also again ran the same analysis for two simulated sets of data that were generated using the dual-systems RL model of the Daw paradigm. For each set, we matched one agent to each of our participants, copying their parameter fits except for the weighting parameter, which was set to *w* = 0 for one set (the model-free set), and *w* = 1 for the other (the model-based set).

In these regression analyses, the main effect of the previous reward (*r*_i-1_) represents the model-free contribution to choice, since it captures reward effects that are independent from the transition type on the previous trial, whereas the whereas the interaction term *r*_i-1_ * *common*_i_ captures reward effects that are modulated by the transition type on the previous trial and therefore represents the model-based contribution to choice. For the experimental data, we computed the difference between the model-based and model-free coefficients as an analogous term to the weighting parameter *w* in the computational model.

Results

*Novel paradigm.* The results from the logistic regression for the novel paradigm are given in Table S3. For the experimental data we found significant effects of the regressors indicating the outcome of the previous trial, indicating a model-based contribution, and the interaction between previous outcome and the similarity of the current and previous first-stage states, indicating the model-free contribution. Importantly, we found no significant interaction between reward and similarity of the current and previous first-stage states for the simulated data of model-based agents, but this interaction was significant for the model-free agents, as expected.

**Table S3.** Regression coefficients from multilevel logistic regression analysis, indicating the effect of outcome of previous trial, similarity of previous starting state to current starting state, and previous difference between chosen and unchosen reward, on repetition of second-stage choice for experimental data and simulated data from pure model-based and pure-model free agents matched to fits from experimental data.

|  | Experimental data | | Model-free agents | | Model-based agents | |
| --- | --- | --- | --- | --- | --- | --- |
| Coefficient | Estimate (SE) | *p* | Estimate (SE) | *p* | Estimate (SE) | *p* |
| (Intercept) | .47 (.04) | < .001 | .42 (.04) | < .001 | .54 (.05) | < .001 |
| Previous reward | .31 (.02) | < .001 | .13 (.01) | < .001 | .23 (.02) | < .001 |
| Same starting state | .21 (.03) | < .001 | .15 (.02) | < .001 | -.00 (.02) | .98 |
| Previous reward difference | .06 (.01) | < .001 | .04 (.01) | < .001 | .08 (.01) | < .001 |
| Reward × Same | .16 (.02) | < .001 | .07 (.01) | < .001 | -.00 (.01) | .71 |
| Reward × Difference | -.00 (.00) | .39 | -.00 (.00) | < .01 | .01 (.00) | < .01 |
| Difference × Same | -.00 (.01) | .98 | .00 (.01) | .79 | .00 (.00) | .97 |
| Reward × Same × Difference | .01 (.00) | .02 | -.00 (.00) | .76 | .00 (.00) | .87 |

*Daw paradigm.* The results from the logistic regression for the Daw paradigm are given in Table S4. For the experimental data we found significant effects of the regressors indicating the outcome of the previous trial, indicating a model-free contribution, and the interaction between previous outcome and previous transition type, indicating the model-free contribution. For the simulated data, we found no significant interaction between reward and transition type for the simulated data of model-free agents and no significant main effect of reward for the simulated data of model-based agents as expected.

**Table S4.** Regression coefficients from multilevel logistic regression analysis, indicating the effect of outcome of previous trial, transition type of previous trial, on repetition of first-stage choice for experimental data and simulated data from pure model-based and pure-model free agents matched to fits from experimental data.

|  | Experimental data | | Model-free agents | | Model-based agents | |
| --- | --- | --- | --- | --- | --- | --- |
| Coefficient | Estimate (SE) | *p* | Estimate (SE) | *p* | Estimate (SE) | *p* |
| (Intercept) | 1.03 (.07) | < .001 | 1.19 (.09) | < .001 | .80 (.06) | < .001 |
| Previous reward | .26 (.03) | < .001 | .33 (.03) | < .001 | .01 (.02) | .54 |
| Previous transition | .03 (.02) | .14 | .00 (.02) | .89 | .03 (.02) | .12 |
| Reward × Transition | .20 (.03) | < .001 | .02 (.02) | .38 | .18 (.02) | < .001 |

*Correlations.* We found that our indices of the relative weighting between model-based and model-free control were positively related to our measure of reward that controlled for average chance performance for the novel task (*r* = 0.69, *p* < 0.001), but not for the Daw paradigm (*r* = 0.03, *p* = 0.71). A subsequent multiple regression showed that this relationship was significantly different between groups [*t*(377) = 7.33, *p* < 0.001]. These results provide convergent evidence for the accuracy-demand trade-off of the novel two-step paradigm, and for the verification of the prediction that the original Daw two-step paradigm does not embody such a trade-off.
