# Supplementary material for: When Does Model-Based Control Pay Off?
Source: PLoS Comput Biol. 2016 Aug 26;12(8):e1005090. doi: 10.1371/journal.pcbi.1005090 (PMC5001643; doi:10.1371/journal.pcbi.1005090)
Supplement: S1 Table — (DOCX) [file pcbi.1005090.s005.docx]

**S1 Table. Model comparison for the full hybrid model and the hybrid model without choice perseveration parameters.**

| Paradigm | Model | Number of parameters (*k)* | Log-likelihood | BIC | AIC | McFadden’s pseudo R^2^ |
| --- | --- | --- | --- | --- | --- | --- |
| Daw | No stickiness | 4 | -27541 | 58886 | 56657 | 0.19 |
|  | *π* | 5 | -26082 | 56920 | 54134 | 0.24 |
|  | *ρ* | 5 | -26569 | 57893 | 55107 | 0.22 |
|  | ***π , ρ*** | **6** | -25086 | **55880** | **52537** | 0.27 |
|  |  |  |  |  |  |  |
| Novel | **No stickiness** | **4** | -11742 | 27038 | 24957 | 0.26 |
|  | *π* | 5 | -11535 | 27511 | 24909 | 0.28 |
|  | *ρ* | 5 | -11273 | **26989** | 24387 | 0.29 |
|  | *π , ρ* | 6 | -11035 | 27401 | **24279** | 0.31 |

Note: The number of trials in both experiments was *n* = 125, and the number of participants in the Daw paradigm *N* = 197, and in the novel paradigm, *N* = 184, and therefore BIC = -2 × Log-likelihood + *k* × *N* × log (*n*), and AIC = 2 × *k* × *N* - 2 × Log-likelihood. McFadden’s pseudo R^2^ is computed as (R - Log-likelihood)/R where R is the log-likelihood for the chance model (125 × 2 × ln (1/2) for the Daw paradigm and 125 × ln (1/2) for the Doll paradigm).
