# Supplementary material for: When Does Model-Based Control Pay Off?
Source: PLoS Comput Biol. 2016 Aug 26;12(8):e1005090. doi: 10.1371/journal.pcbi.1005090 (PMC5001643; doi:10.1371/journal.pcbi.1005090)
Supplement: S2 Table — (DOCX) [file pcbi.1005090.s006.docx]

**S2 Table. Model comparison for the hybrid model and pure model-based and model-free models.**

| Paradigm | Model | Number of parameters (*k)* | Log-likelihood | BIC | AIC | McFadden’s pseudo R^2^ |
| --- | --- | --- | --- | --- | --- | --- |
| Daw | Hybrid | 6 | -25086 | 55880 | **52537** | 0.27 |
|  | Model-free | 5 | -25346 | **55449** | 52663 | 0.26 |
|  | Model-based | 5 | -25561 | 55878 | 53092 | 0.25 |
|  |  |  |  |  |  |  |
| Novel | **Hybrid** | 5 | -11273 | **26989** | **24387** | 0.29 |
|  | Model-free | 4 | -12173 | 27900 | 25818 | 0.24 |
|  | Model-based | 4 | -11804 | 27162 | 25080 | 0.26 |

Note: The number of trials in both experiments was *n* = 125, and the number of participants in the Daw paradigm *N* = 197, and in the novel paradigm, *N* = 184, and therefore BIC = -2 × ln (Likelihood) + *k* × *N* × ln (*n*), and AIC = 2 × *k* × *N* - *2* × ln (Likelihood). McFadden’s pseudo R^2^ is computed as (R - ln (Likelihood))/R where R is the log-likelihood for the chance model (125 × 2 × ln (1/2) for the Daw paradigm and 125 × ln (1/2) for the Doll paradigm).
